# Hepatic PTP4A1 ameliorates high-fat diet-induced hepatosteatosis and hyperglycemia by the activation of the CREBH/FGF21 axis

**DOI:** 10.7150/thno.79434

**Published:** 2023-01-22

**Authors:** Byungtae Hwang, Min-Gi Kwon, Min Ji Cho, Nam-Kyung Lee, Jangwook Lee, Jeong Woong Lee, Kyoung-Jin Oh, Kwang-Hee Bae, Jung Hwan Hwang, Jeong-Ki Min, Jong-Gil Park

**Affiliations:** 1 Biotherapeutics Translational Research Center, Korea Research Institute of Bioscience & Biotechnology (KRIBB), 125 Gwahak-ro, Yuseong-gu, Daejeon 34141, Republic of Korea.; 2 Metabolic Regulation Research Center, Korea Research Institute of Bioscience & Biotechnology (KRIBB), 125 Gwahak-ro, Yuseong-gu, Daejeon 34141, Republic of Korea.; 3 Laboratory Animal Resource Center, Korea Research Institute of Bioscience & Biotechnology (KRIBB), 125 Gwahak-ro, Yuseong-gu, Daejeon 34141, Republic of Korea.; 4 Department of Bioscience, KRIBB School of Bioscience, Korea University of Science and Technology (UST), 125 Gwahak-ro, Yuseong-gu, Daejeon 34141, Republic of Korea.

**Keywords:** PTP4A1, hepatosteatosis, glucose homeostasis, CREBH, FGF21

## Abstract

Precise regulation of kinases and phosphatases is crucial for human metabolic homeostasis. This study aimed to investigate the roles and molecular mechanisms of protein tyrosine phosphatase type IVA1 (PTP4A1) in regulating hepatosteatosis and glucose homeostasis.

**Method:** *Ptp4a1^-/-^* mice, adeno-associated virus encoding *Ptp4a1* under liver-specific promoter, adenovirus encoding *Fgf21*, and primary hepatocytes were used to evaluate PTP4A1-mediated regulation in the hepatosteatosis and glucose homeostasis. Glucose tolerance test, insulin tolerance test, 2-deoxyglucose uptake assay, and hyperinsulinemic-euglycemic clamp were performed to estimate glucose homeostasis in mice. The staining, including oil red O, hematoxylin & eosin, and BODIPY, and biochemical analysis for hepatic triglycerides were performed to assess hepatic lipids. Luciferase reporter assays, immunoprecipitation, immunoblots, quantitative real-time polymerase chain reaction, and immunohistochemistry staining were conducted to explore the underlying mechanism.

**Results:** Here, we found that deficiency of PTP4A1 aggravated glucose homeostasis and hepatosteatosis in mice fed a high-fat (HF) diet. Increased lipid accumulation in hepatocytes of *Ptp4a1^-/-^* mice reduced the level of glucose transporter 2 on the plasma membrane of hepatocytes leading to a diminution of glucose uptake. PTP4A1 prevented hepatosteatosis by activating the transcription factor cyclic adenosine monophosphate-responsive element-binding protein H (CREBH)/fibroblast growth factor 21 (FGF21) axis. Liver-specific PTP4A1 or systemic FGF21 overexpression in *Ptp4a1^-/-^* mice fed an HF diet restored the disorder of hepatosteatosis and glucose homeostasis. Finally, liver-specific PTP4A1 expression ameliorated an HF diet-induced hepatosteatosis and hyperglycemia in wild-type mice.

**Conclusions:** Hepatic PTP4A1 is critical for regulating hepatosteatosis and glucose homeostasis by activating the CREBH/FGF21 axis. Our current study provides a novel function of PTP4A1 in metabolic disorders; hence, modulating PTP4A1 may be a potential therapeutic strategy against hepatosteatosis-related diseases.

## Introduction

The protein tyrosine phosphatase type IVA 1/phosphatase of regenerating liver-1 (PTP4A1/PRL-1), which belongs to dual-specificity phosphatases, is localized in the nucleus, peri-nucleus, and plasma membrane involved in various intracellular signaling 1-4. PTP4A1 consists of phosphatase site, polybasic region, and prenylation motif and promotes cell proliferation, migration, and invasion through regulating the expression and/or activity of cell cycle regulators, p53, focal adhesion complex proteins, Rho and extracellular signal-regulated kinase signaling cascades 5-7. PTP4A1 directly interacts with some molecules, including phospholipids, activating transcription factor 5/7 (ATF5/7), and p115 Rho GTPase activating protein for regulating their activities 6, 8. Initially, PTP4A1 has identified as an immediate-early gene because PTP4A1 is highly expressed during liver regeneration 9. Hepatic PTP4A1 deficiency delays liver mass restoration after partial hepatectomy through the impaired phosphoinositide 3-kinase/protein kinase B (PKB, also known as Akt) signaling in PTP4A1 mutant mice 10. However, it remains to be elucidated whether PTP4A1 directly regulates hepatic metabolic diseases, including hepatosteatosis and glucose homeostasis.

Hepatosteatosis is a metabolic disorder with an accumulation of fat in the liver of at least 5% more, which is a clinical hallmark of non-alcoholic fatty liver disease (NAFLD) 11, 12. NAFLD is the most common liver disorder worldwide and is an expanding health problem estimating a global prevalence of 25% 13. NAFLD is categorized into non-alcoholic fatty liver and non-alcoholic steatohepatitis, which are histologically distinguished by inflammation with hepatocyte ballooning injury 14. As the liver is an essential organ for maintaining glucose homeostasis, NAFLD is closely associated with the dysregulation of glucose uptake and utilization by the liver, accompanying the augmentation of gluconeogenesis and impairment of hepatic glucose transporter 2 (GLUT2) translocation to the plasma membrane 15-18. Previous studies prove that NAFLD is associated with liver-related mortality or morbidity and an increased risk of extrahepatic diseases; however, there are currently no approved therapeutics 19. Early development of therapeutics for NAFLD has been focused on preventing the pathogenesis of fibrosis, which is closely linked to the progression of NAFLD to cirrhosis and hepatocellular carcinoma 20. Because of limited clinical efficacy in anti-fibrotic therapeutics, the approaches for developing therapeutics against NAFLD have been replaced with the repression of hepatosteatosis 21, 22. Fibroblast growth factor 21 (FGF21), a hormone expressed primarily by the liver, has been considered a promising therapeutic agent in decreasing hepatosteatosis and hepatocyte injury 21, 23. Administration of FGF21 protein, FGF21 analogs, or delivery of FGF21 by adenovirus into rodent models of NAFLD prevented hepatosteatosis with the suppression of *de novo* lipogenesis and the increased fat oxidation in the liver 24-27. In addition, FGF21, as an endocrine factor, exerts inhibitory effects against NAFLD by regulating diverse extrahepatic tissues, including adipose tissue, pancreas, and skeletal muscle 24-28. FGF21 expression is regulated by changes in a nutritional state, including fasting, high-carbohydrate diets, or low-protein diets through the activation of peroxisome proliferator-activated receptor (PPAR) α and/or cyclic adenosine monophosphate-responsive element-binding protein H (CREBH) 27, 29.

Here, we identified PTP4A1 as a negative regulator in the pathogenesis of hepatic steatosis through activation of the CREBH/FGF21 axis. Deficiency of PTP4A1 in mice accelerates hepatic steatosis in mice fed a high-fat (HF) diet and leads to the disruption of glucose homeostasis with the reduction of GLUT2 on the plasma membrane of hepatocytes. PTP4A1 prevented hepatosteatosis through the activation of the transcription factor CREBH/FGF21 axis. Liver-specific PTP4A1 or systemic FGF21 overexpression in *Ptp4a1^-/-^* mice fed an HF diet restored PTP4A1 deficiency-mediated upregulation of hepatosteatosis and blood glucose. In addition, liver-specific PTP4A1 expression ameliorated HF diet-induced hepatosteatosis and hyperglycemia in wild-type (WT) mice. Therefore, controlling PTP4A1 may be a potential candidate for therapeutic strategy against hepatosteatosis-related diseases.

## Results

### The deficiency of PTP4A1 in mice exacerbates HF diet-induced hyperglycemia and NAFLD

The mRNA levels of *Ptp4a1*, but not other isoforms, were significantly increased in the liver of mice fed an HF diet compared to those of mice fed a normal chow (NC) diet **(Figure S1A)**. Also, the alteration of hepatic fat contents by fasting and refeeding increased the *Ptp4a1* mRNA levels in mice fed an NC diet **(Figure S1B)**. Consistent with mouse data, human hepatoma cells treated with bovine serum albumin-oleic acid (BSA-OA) tended to increase *PTP4A1* mRNA levels compared to controls **(Figure S1C)**. In addition, we identified that the levels of *PTP4A1* expression were significantly increased in human NASH samples than in healthy controls in the dataset (GSE63067) from the Gene Expression Omnibus database **(Figure S1D)**.

**Figure 1:**
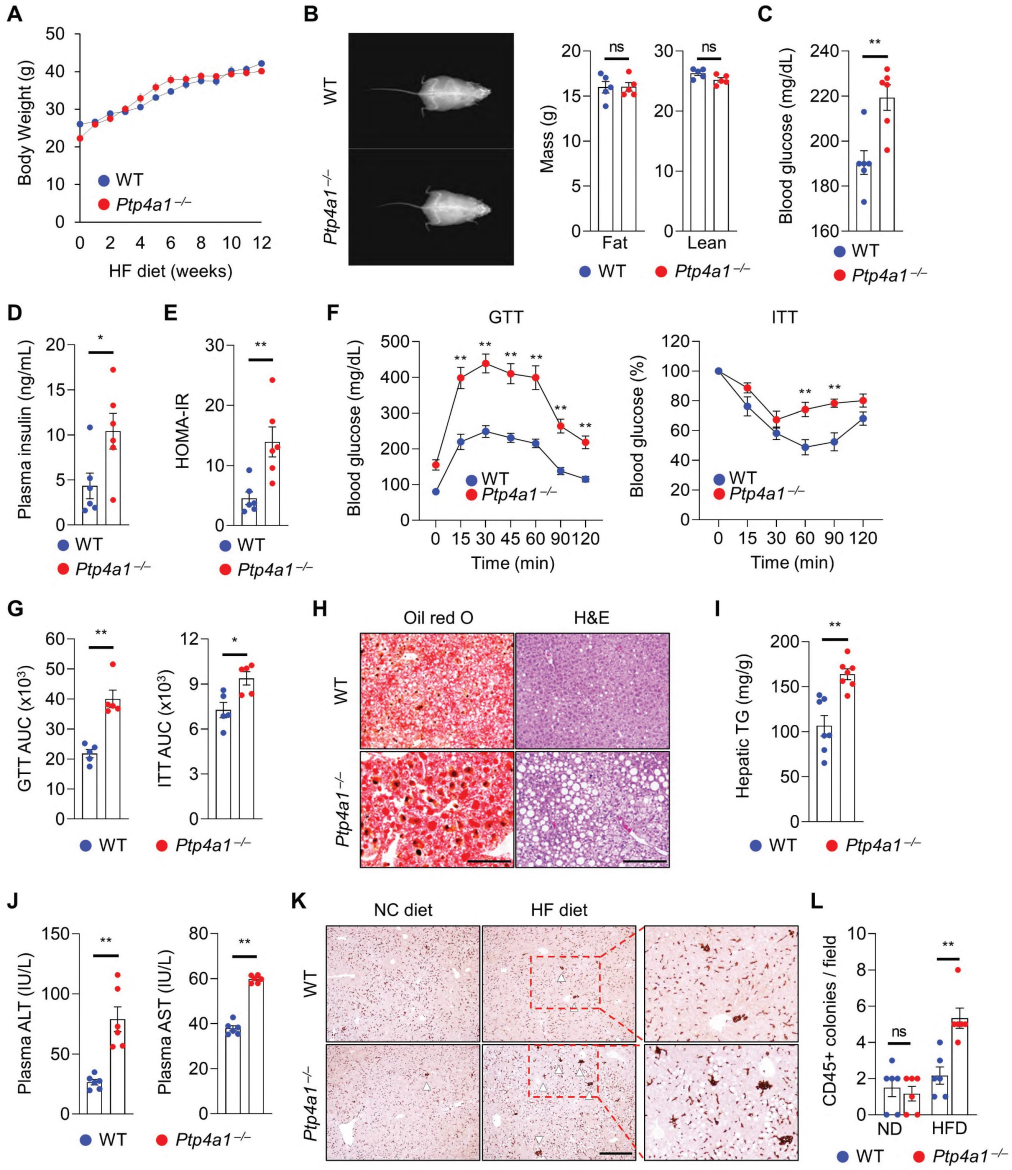
** Lacking PTP4A1 in mice exacerbates a high-fat (HF) diet-induced hyperglycemia and NAFLD. (A)** Body weight (BW) gain of *Ptp4a1^-/-^* mice and wild-type (WT) littermates on an HF diet for 12 weeks (n = 7). **(B)** Representative images and the graphs for fat and lean mass in dual-energy X-ray absorptiometry analysis (n = 5). **(C and D)** The levels of blood glucose (C) and plasma insulin (D) of WT and *Ptp4a1^-/-^* mice fed an HF diet for 12 weeks (n = 6). **(E)** Homeostatic model assessment-insulin resistance (HOMA-IR) of WT and *Ptp4a1^-/-^* mice in fasting conditions (n = 6). **(F)** Glucose tolerance test (GTT) and insulin tolerance test (ITT) of WT and *Ptp4a1^-/-^* mice fed an HF diet for 12 weeks (n = 5). **(G)** The area under the curve (AUC) of GTT and ITT (n = 5). **(H)** The representative images for oil-red O and hematoxylin & eosin (H&E) staining in the livers from WT and *Ptp4a1^-/-^* mice fed an HF diet for 12 weeks (n = 7). Scale bar, 200 μm. **(I)** Hepatic triglyceride (TG) levels of WT and *Ptp4a1^-/-^* mice fed an HF diet for 12 weeks (n = 7). **(J)** The levels of alanine aminotransferase (ALT) and aspartate transaminase (AST) in plasma of WT and *Ptp4a1^-/-^* mice fed an HF diet for 12 weeks (n = 6). **(K)** The representative images for CD45 positive cells in the liver of WT and *Ptp4a1^-/-^* mice fed a normal chow (NC) or an HF diet (n = 6). Scale bar, 300 μm. The magnified images in the red dot boxes are presented on the right. **(L)** The quantitative graph for CD45 positive colonies per field in the liver of WT and *Ptp4a1^-/-^* mice fed an HF diet for 12 weeks (n = 6). Data are presented as the mean ± standard error of the mean. *P < 0.05, **P < 0.01, n.s., not significant (Mann-Whitney U test for B-E, G, I, and J, two-way ANOVA for F and L).

To evaluate whether PTP4A1 affects the regulation of obese-mediated metabolic diseases such as type 2 diabetes and NAFLD, we generated *Ptp4a1^-/-^* mice by CRISPR/Cas9 system **(Figure S2)** and placed *Ptp4a1^-/-^* mice and WT littermates on an HF diet for 12 weeks. Body weight (BW) gain and food intake were comparable between the two groups on an HF diet for 12 weeks **(Figure 1A and S3A)**. Dual-energy X-ray absorptiometry (DEXA) revealed that both lean and fat mass were similar between the two groups **(Figure 1B)**, and the ratio of epididymal white adipose tissue (eWAT) mass to BW was also comparable between the two groups **(Figure S3B)**. However, the blood glucose levels in *Ptp4a1^-/-^* mice were significantly higher than in WT mice in a postprandial state after 12 weeks of an HF diet feeding **(Figure 1C)**. Concomitantly, plasma insulin levels were markedly increased in *Ptp4a1^-/-^* mice compared to WT mice fed an HF diet **(Figure 1D)**. In fasting conditions, homeostatic model assessment-insulin resistance (HOMA-IR) was significantly increased in *Ptp4a1^-/-^* mice compared to WT mice after an HF diet **(Figure 1E)**. Glucose tolerance test (GTT) and insulin tolerance test (ITT) revealed that deficiency of PTP4A1 in mice fed an HF diet exacerbated glucose tolerance and reduced insulin sensitivity **(Figure 1F-G)**. After an HF diet for 12 weeks, *Ptp4a1^-/-^* mice showed an increase in fat accumulation in the liver compared to those of WT mice, which was proved by oil red O staining, hematoxylin and eosin (H&E) staining, and hepatic triglyceride (TG) analysis **(Figure 1H-I)**. In addition, *Ptp4a1^-/-^* mice increased the levels of alanine aminotransferase (ALT) and aspartate transaminase (AST) in plasma compared to those of WT mice fed an HF diet **(Figure 1J)**. By immunohistochemistry assay for cluster of differentiation 45 (CD45), we verified the enhanced immune cell accumulation in the liver of *Ptp4a1^-/-^* mice compared to WT mice fed an HF diet **(Figure 1K-L)**. Consistently, inflammatory markers, including *F4/80*, *Mcp1*, and *Mip1a*, but not *Cd11c* and* KC,* were significantly increased in the liver of *Ptp4a1^-/-^* mice compared to WT mice fed an HF diet **(Figure S3C)**.

### Lack of PTP4A1 reduces glucose uptake by decreasing GLUT2 on the plasma membrane in hepatocytes

**Figure 2:**
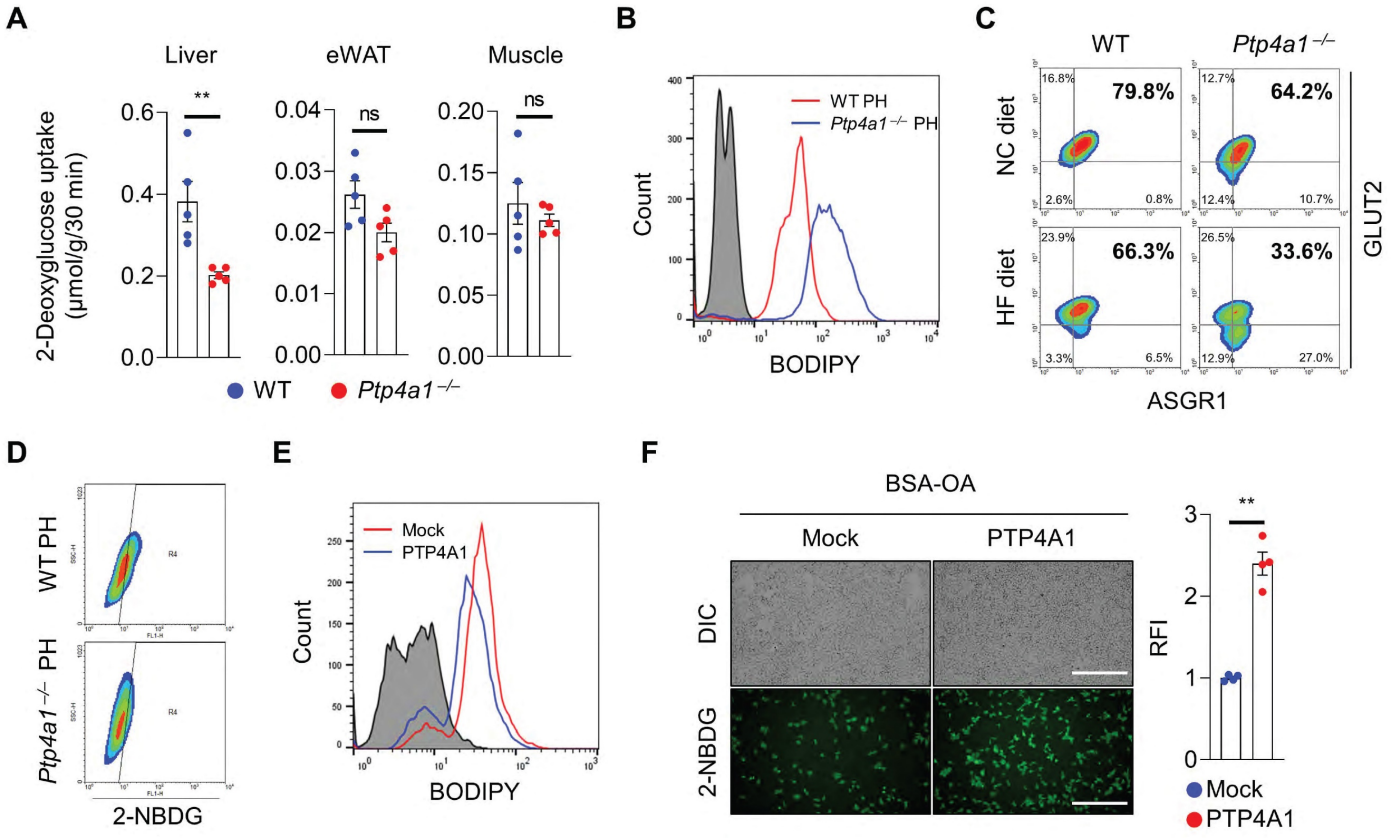
** The deficiency of PTP4A1 in mice fed a high-fat (HF) diet reduces glucose uptake by a decrease in GLUT2 on the plasma membrane in hepatocytes. (A)** The assay of 2-deoxyglucose (2-DG) uptake in the livers, epididymal white adipose tissues (eWAT), and gastrocnemius muscles of wild-type (WT) and *Ptp4a1^-/-^* mice fed an HF diet for 12 weeks (n = 5).** (B)** FACS analysis after staining BODIPY on primary hepatocytes (PH) of WT and *Ptp4a1^-/-^* mice fed an HF diet. Data represent three independent experiments. **(C)** FACS analysis after staining glucose transporter 2 (GLUT2)-APC and asialoglycoprotein receptor 1 (ASGR1)-Alexa 488 on PH of WT and *Ptp4a1^-/-^* mice fed a normal chow (NC) or an HF diet. ASGR1 was used as a marker for hepatocytes. Data represent three independent experiments.** (D)** FACS analysis of the 2-NBDG glucose uptake assay on the PH of WT and *Ptp4a1^-/-^* mice fed an HF diet. Data represent three independent experiments. **(E)** FACS analysis after staining BODIPY on Hep3B expressing mock or PTP4A1 treated with bovine serum albumin-oleic acid (BSA-OA). Data represent three independent experiments. **(F)** The 2-NBDG uptake assay after incubation of BSA-OA on Hep3B transfected by PTP4A1-expressing vector or control vector. Representative images (left) and quantification for relative fluorescence intensity (RFI, right) (n = 4). Scale bar, 500 μm. Data are presented as the mean ± standard error of the mean. **P < 0.01, n.s., not significant (Mann-Whitney U test for A; two‐tailed Student's t‐test for F).

To verify whether the alteration of gluconeogenesis contributes to an increase in blood glucose in *Ptp4a1^-/-^* mice fed an HF diet compared to controls, we performed the pyruvate tolerance test (PTT) and glycerol tolerance test (GlyTT) in WT and *Ptp4a1^-/-^* mice fed an HF diet. After starvation, both groups were injected with pyruvate or glycerol as a substrate for glucose production. The blood glucose levels were comparable between the two groups in PTT and GlyTT analyses **(Figure S4A-B)**. In addition, the mRNA levels of gluconeogenesis, including *Foxo1*, *Pck1*, and *G6pc,* were comparable between the two groups **(Figure S4C)**. Next, we performed the hyperinsulinemic-euglycemic clamp study and 2-deoxyglucose (2-DG) uptake assay in WT and *Ptp4a1^-/-^* mice fed an HF diet. Basal hepatic glucose production (HGP), clamp HGP, and glucose infusion rate were comparable between the two groups; however, glucose uptake tended downward in *Ptp4a1^-/-^* mice compared to WT fed an HF diet in the hyperinsulinemic-euglycemic clamp study **(Figure S4D)**. In the 2-DG uptake assay, we verified that deficiency of PTP4A1 in mice fed an HF diet decreased the levels of 2-DG in livers but not in eWATs and skeletal muscles **(Figure 2A)**.

As hepatic steatosis induces a decrease in plasma membrane GLUT2 levels in the liver of mice fed an HF diet has been reported 16, we verified whether the levels of GLUT2 on the plasma membrane of hepatocytes were reduced by feeding an HF diet compared to an NC diet in mice. Primary hepatocytes revealed that plasma membrane GLUT2 was markedly decreased in the hepatocytes of mice fed an HF diet compared to those fed an NC diet **(Figure S4E)**. In line with increased hepatic TG levels in *Ptp4a1^-/-^* mice fed an HF diet, isolated primary hepatocytes of *Ptp4a1^-/-^* mice showed a higher lipid accumulation than those of WT mice after feeding an HF diet **(Figure 2B and S4F)**. Along with the increased lipid accumulation, deficiency of PTP4A1 in hepatocytes of mice fed an HF diet lessened the levels of GLUT2 on the plasma membrane **(Figure 2C and S4F)** and the uptake of 2-(N-(7-Nitrobenz-2-oxa-1,3-diazol-4-yl)Amino)-2-Deoxyglucose (2-NBDG), a fluorescent glucose analog **(Figure 2D)**. In addition, an inverse correlation between fat accumulation and surface GLUT2 levels in hepatocytes was verified **(Figure S4G)**. Overexpression of PTP4A1 in Hep3B reduced the lipid accumulation after incubation with BSA-OA compared to controls, leading to augmentation of the uptake of 2-NBDG more than controls **(Figure 2E-F)**. In contrast, PTP4A1 down-regulation in Hep3B by *PTP4A1*-specific short hairpin RNA (shRNA)-expressing lentivirus decreased the uptake of 2-NBDG after incubation with BSA-OA compared to controls **(Figure S4H)**.

### PTP4A1 regulates the expression of FGF21 through an increase in CREBH activity

**Figure 3:**
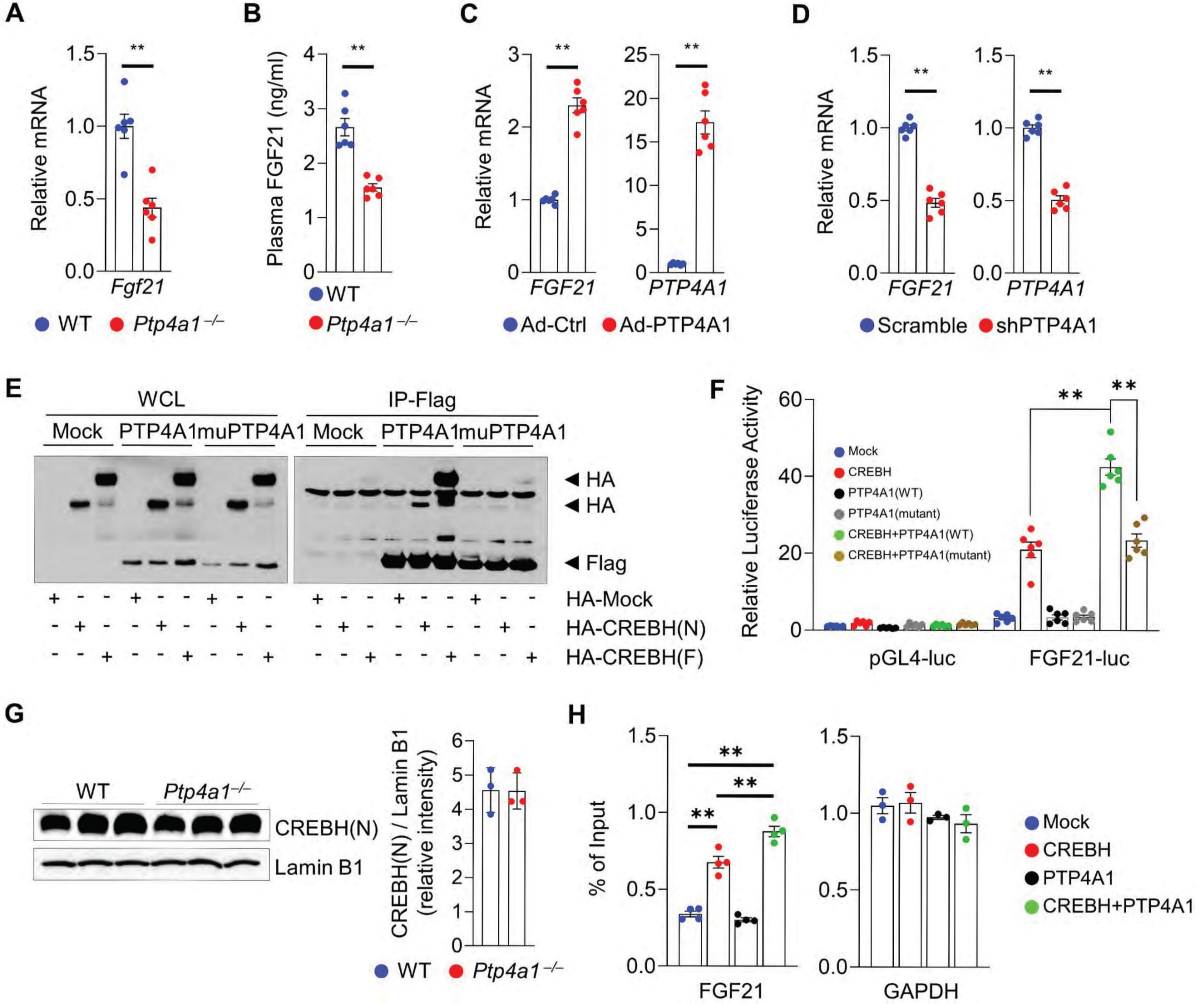
** PTP4A1 regulates the expression of FGF21 via the activation of transcription factor CREBH. (A)** The mRNA levels of *Fgf21* in the liver of wild-type (WT) and *Ptp4a1^-/-^* mice fed an HF diet for 12 weeks (n = 6). **(B)** Plasma FGF21 levels in WT and *Ptp4a1^-/-^* mice fed an HF diet for 12 weeks (n = 6). **(C)** The mRNA levels of *FGF21* and *PTP4A1* in Hep3B infected by adenovirus (Ad)-Control (Ctrl) or Ad-*PTP4A1* (n = 6). **(D)** The mRNA levels of *FGF21* and *PTP4A1* in Hep3B infected by lentivirus expressing-shPTP4A1 or -scramble control (n = 6). **(E)** Co-immunoprecipitation assay in HEK 293T transfected by HA-Mock, HA-CREBH(N), or HA-CREBH(F) with Flag-Mock, Flag-PTP4A1, or Flag-mutant PTP4A1 (D74A/C104S). Flag antibody was used for immunoprecipitation. Data represent three independent experiments. **(F)** FGF21-luciferase reporter assay in HEK293T transfected by Mock, CREBH(N), PTP4A1, mutant PTP4A1, CREBH(N)+PTP4A1, or CREBH(N)+mutant PTP4A1. **(G)** Western blot analysis for the CREBH(N) in the nuclear fraction of liver from WT and *Ptp4a1^-/-^* mice fed an HF diet. Lamin B1 was used for loading control. The quantification graph is presented on the right (n = 3). **(H)** Chromatin immunoprecipitation assay (n = 4). The *FGF21* gene was amplified by specific primers after immunoprecipitation by an anti-HA antibody in mock-, HA-CREBH-, PTP4A1-, or HA-CREBH+PTP4A1-treated Hep3B. *GAPDH* was used as an internal control after immunoprecipitation by an anti-RNA polymerase II antibody. Data represent three independent experiments. Data are presented as the mean ± standard error of the mean. **P < 0.01 (two‐tailed Student's t‐test for A, C, D, and G; Mann-Whitney U test for B; two-way ANOVA for F; one-way ANOVA for H).

To investigate how PTP4A1 deficiency in mice induces hepatic steatosis, we analyzed the levels of transcripts involved in lipid metabolisms. The mRNA levels involved in fatty acids oxidation-related genes such as *Pparα*, *Acox1*, *Cpt1a*, *Cpt2*, *Acadvl*, and *Acadm* were significantly decreased; however, the mRNA levels associated with lipogenic genes were not increased in the liver of *Ptp4a1^-/-^* mice fed an HF diet compared to controls **(Figure S5A-B)**. Interestingly, the mRNA levels of *Fgf21* in the liver of *Ptp4a1^-/-^* mice fed an HF diet were significantly mitigated, and plasma FGF21 levels were also diminished in *Ptp4a1^-/-^* mice fed an HF diet compared to WT mice **(Figure 3A-B)**. Adenoviral overexpression of PTP4A1 in human hepatoma cells increased the mRNA levels of *FGF21* compared to controls **(Figure 3C)**, and shRNA-mediated PTP4A1 knock-down in human hepatoma cells reduced the mRNA levels of *FGF21* compared to controls **(Figure 3D)**. As FGF21 expression is regulated by transcription factor PPARα and/or CREBH, we tested whether PTP4A1 physically interacts with PPARα or CREBH in human embryonic kidney 293T (HEK293T) cells. By co-immunoprecipitation, we identified that PTP4A1 physically interacted with the active form of CREBH (CREBH(N)) and the inactive form of CREBH (CREBH(F)) but not PPARα **(Figure 3E and S5C)**. PTP4A1 synergistically increased CREBH-mediated luciferase activity in an FGF21-luciferase reporter assay **(Figure 3F)**. However, mutation of PTP4A1 at the residues of 72 (D to A) and 104 (C to S) amino acids did not increase CREBH-mediated luciferase activity in an FGF21-luciferase reporter assay because mutant of PTP4A1 failed to interact with CREBH **(Figure 3E-F)**. To verify the molecular mechanism for the modulatory role of PTP4A1 in the CREBH-FGF21 axis, we first confirmed the levels of CREBH in the liver of WT and *Ptp4a1^-/-^* mice. The mRNA levels of the *Creb3l3* gene (encoding CREBH) were comparable between the liver of WT and *Ptp4a1^-/-^* mice **(Figure S5D)**. To confirm the protein levels of CREBH(N) in the liver, we isolated the nuclear fraction and identified the similar levels of CREBH(N) between the two groups **(Figure 3G)**. Next, we tested whether PTP4A1 may increase the CREBH binding activity on the *FGF21* promoter region by chromatin immunoprecipitation (ChIP) assay. Consistent with the luciferase reporter gene assay, we verified that PTP4A1 could enhance the activity of CREBH on the FGF21 gene expression **(Figure 3H)**.

### PTP4A1 deficiency in mice disturbs FGF21 expression, blood glucose, and hepatic TG in fasting conditions

**Figure 4:**
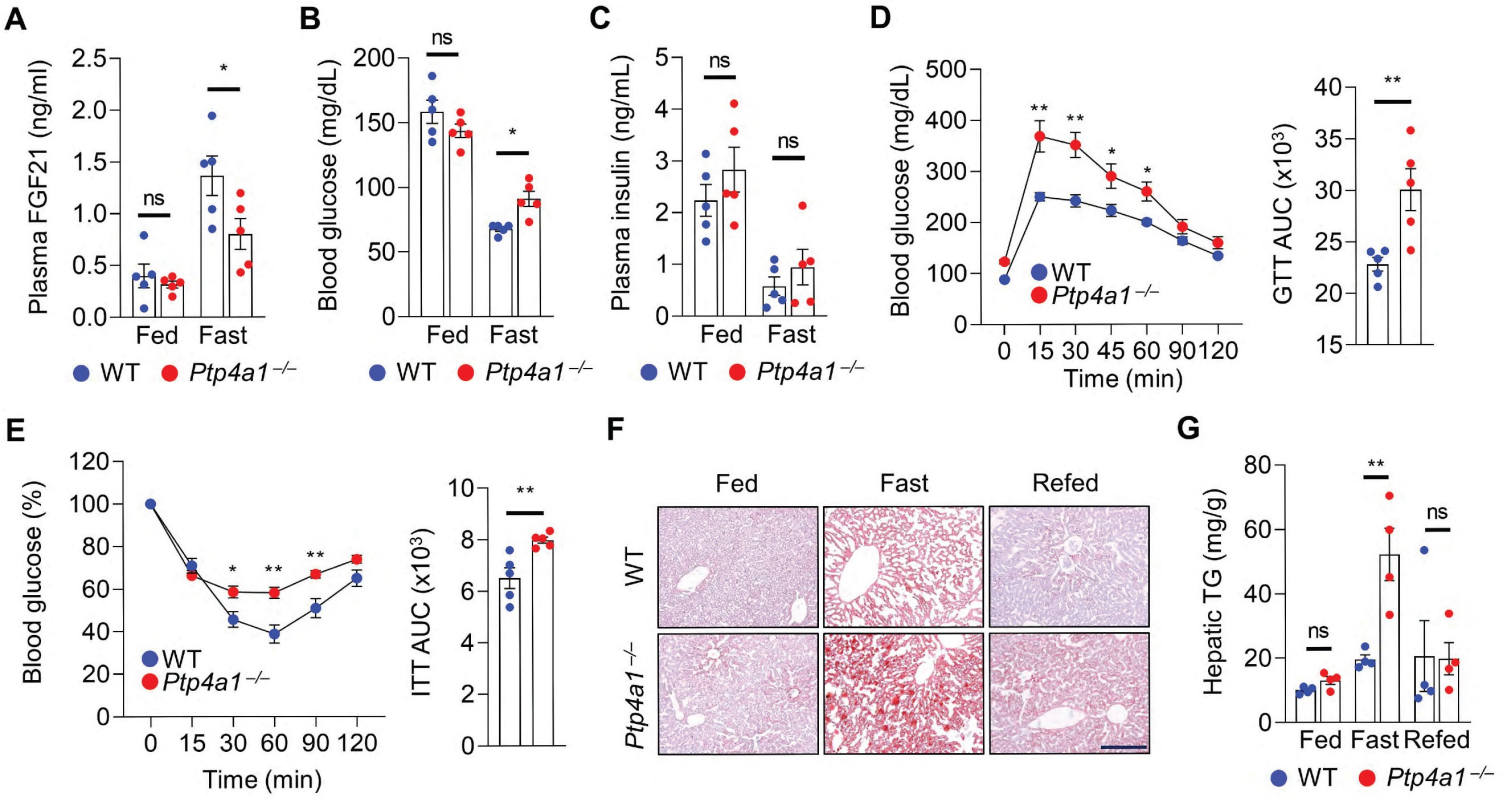
** Lacking PTP4A1 in mice increases blood glucose and NAFLD by the down-regulation of FGF21 expression in fasting conditions. (A)** Plasma FGF21 levels in wild-type (WT) and *Ptp4a1^-/-^* mice on a normal chow (NC) diet in feeding and fasting conditions (n = 5). **(B and C)** The blood glucose levels (B) and plasma insulin levels (C) in WT and *Ptp4a1^-/-^* mice on an NC diet in feeding and fasting conditions (n = 5). **(D)** Glucose tolerance test (GTT) and the area under the curve (AUC) of GTT in WT and *Ptp4a1^-/-^* mice fed an NC diet (n = 5). **(E)** Insulin tolerance test (ITT) and the AUC of ITT in WT and *Ptp4a1^-/-^* mice fed an NC diet (n = 5). **(F)** The representative images for oil red O staining in the liver sections of WT and *Ptp4a1^-/-^* mice fed an NC diet in feeding, fasting, and refeeding conditions (n = 4). Scale bar, 200 μm. **(G)** Hepatic triglyceride (TG) of WT and *Ptp4a1^-/-^* mice fed an NC diet in feeding, fasting, and refeeding conditions (n = 4). Data are presented as the mean ± standard error of the mean. *P < 0.05, **P < 0.01, n.s., not significant (two-way ANOVA for A-C, D (left), E (left) and G; Mann-Whitney U test for D (right) and E (right)).

As FGF21 expression is induced by fasting, we investigated the levels of FGF21 in WT and *Ptp4a1^-/-^* mice after feeding and fasting. The plasma FGF21 levels were comparable between two groups fed an NC diet; however, those were significantly mitigated in *Ptp4a1^-/-^* mice after fasting compared to WT mice **(Figure 4A)**. The blood glucose levels were higher in *Ptp4a1^-/-^* mice than WT mice after fasting but not fed an NC diet **(Figure 4B)**. However, plasma insulin levels of the two groups were comparable **(Figure 4C)**. After fasting, *Ptp4a1^-/-^* mice revealed impaired glucose tolerance and insulin sensitivity by GTT and ITT, respectively **(Figure 4D-E)**. Consistently, the levels of hepatic TG were significantly augmented in *Ptp4a1^-/-^* mice compared to WT mice by fasting; however, those were comparable between the two groups after feeding and refeeding an NC diet **(Figure 4F-G)**. During feeding an NC diet from 8 to 22 weeks old, the BW of WT and *Ptp4a1^-/-^* mice were comparable, and the ratios of eWAT/BW and liver/BW were not different **(Figure S6A-C)**.

### Liver-specific PTP4A1 or systemic FGF21 overexpression ameliorates hyperglycemia and NAFLD in *Ptp4a1^-/-^* mice fed an HF diet

**Figure 5:**
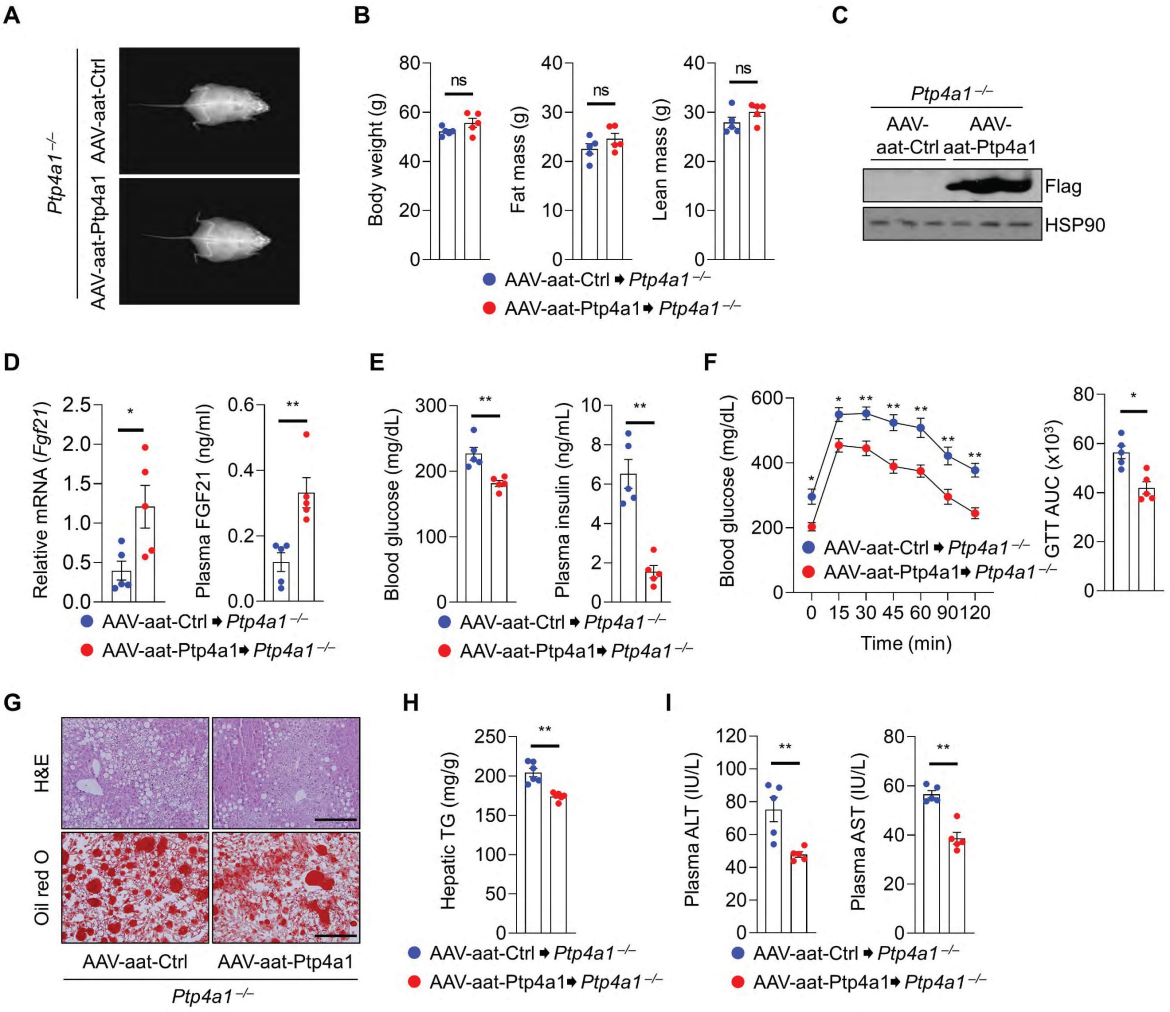
** Liver-specific PTP4A1 overexpression ameliorates hyperglycemia and NAFLD in *Ptp4a1^-/-^* mice fed a high-fat (HF) diet. (A and B)** Representative images **(A)** and the graphs **(B)** for body weight, fat, and lean mass of ***Ptp4a1****^-/-^* mice administrated with adeno-associated virus (AAV)-aat-control (Ctrl) or AAV-aat-*Ptp4a1* after feeding an HF diet for 12 weeks in dual-energy X-ray absorptiometry analysis **(n = 5)**. **(C)** Immunoblot analysis in the liver lysates of *Ptp4a1^-/-^* mice administrated with AAV-aat-Ctrl or AAV-aat-*Ptp4a1*. HSP90 was used as a loading control. **(D)** The levels of *Fgf21* mRNA in the liver and FGF21 in plasma of *Ptp4a1^-/-^* mice administrated with AAV-aat-Ctrl or AAV-aat-*Ptp4a1* after feeding an HF diet (n = 5). **(E)** Blood glucose levels (left) and plasma insulin levels (right) in *Ptp4a1^-/-^* mice administrated with AAV-aat-Ctrl or AAV-aat-*Ptp4a1* after feeding an HF diet (n = 5). **(F)** Glucose tolerance test (GTT) and the area under the curve (AUC) of GTT in *Ptp4a1^-/-^* mice administrated with AAV-aat-Ctrl or AAV-aat-*Ptp4a1* after feeding an HF diet (n = 5). (G) The representative images for hematoxylin&eosin (H&E) staining and oil red O staining in the liver sections of *Ptp4a1^-/-^* mice administrated with AAV-aat-Ctrl or AAV-aat-*Ptp4a1* after feeding an HF diet (n = 4). Scale bar, 200 μm. (H) Hepatic triglyceride (TG) levels in *Ptp4a1^-/-^* mice administrated with AAV-aat-Ctrl or AAV-aat-*Ptp4a1* after feeding an HF diet (n = 6). (I) The levels of alanine aminotransferase (ALT) and aspartate transaminase (AST) in plasma of *Ptp4a1^-/-^* mice administrated with AAV-aat-Ctrl or AAV-aat-*Ptp4a1* after feeding an HF diet (n = 5). Data are presented as the mean ± standard error of the mean. *P < 0.05, **P < 0.01, n.s., not significant (Mann-Whitney U test for B, D (right), E, F (right), H, and I; two‐tailed Student's t‐test for D (left); two-way ANOVA for F (left)).

To evaluate the role of PTP4A1 in the liver tissue, *Ptp4a1^-/-^* mice were administrated with adeno-associated virus (AAV)-aat-control (Ctrl) or AAV-aat-*Ptp4a1* under the control of the liver-specific promoter alpha-1-antitrypsin (aat) and were fed an HF diet for 12 weeks **(Figure S7A)**. The liver-specific expression of PTP4A1 in mice injected by AAV-aat-*Ptp4a1* was validated by western blot analysis for Flag-tag **(Figure S7B)**. After an HF diet for 12 weeks, *Ptp4a1^-/-^* mice injected AAV-aat-Ctrl and AAV-aat-*Ptp4a1* showed similar BW, fat mass, and lean mass **(Figure 5A-B)**. PTP4A1 in the liver of *Ptp4a1^-/-^* mice injected with AAV-aat-*Ptp4a1* was highly expressed **(Figure 5C)**, and the mRNA levels of hepatic *Fgf21* and plasma FGF21 levels were increased **(Figure 5D)**. AAV-aat-*Ptp4a1* administration diminished blood glucose and plasma insulin levels compared to AAV-aat-Ctrl in *Ptp4a1^-/-^* mice fed an HF diet for 12 weeks **(Figure 5E)**. The glucose tolerance was markedly improved, and the insulin sensitivity was slightly enhanced by administration of AAV-aat-*Ptp4a1* in *Ptp4a1^-/-^* mice fed an HF diet **(Figure 5F and S7C)**. By histological analyses, we verified that AAV-aat-*Ptp4a1* significantly reduced lipid accumulation in the liver of *Ptp4a1^-/-^* mice fed an HF diet **(Figure 5G-H)**. Finally, the decreased levels of ALT and AST in plasma of *Ptp4a1^-/-^* mice injected with AAV-aat-*Ptp4a1* were identified compared to controls after feeding an HF diet for 12 weeks **(Figure 5I)**.

**Figure 6:**
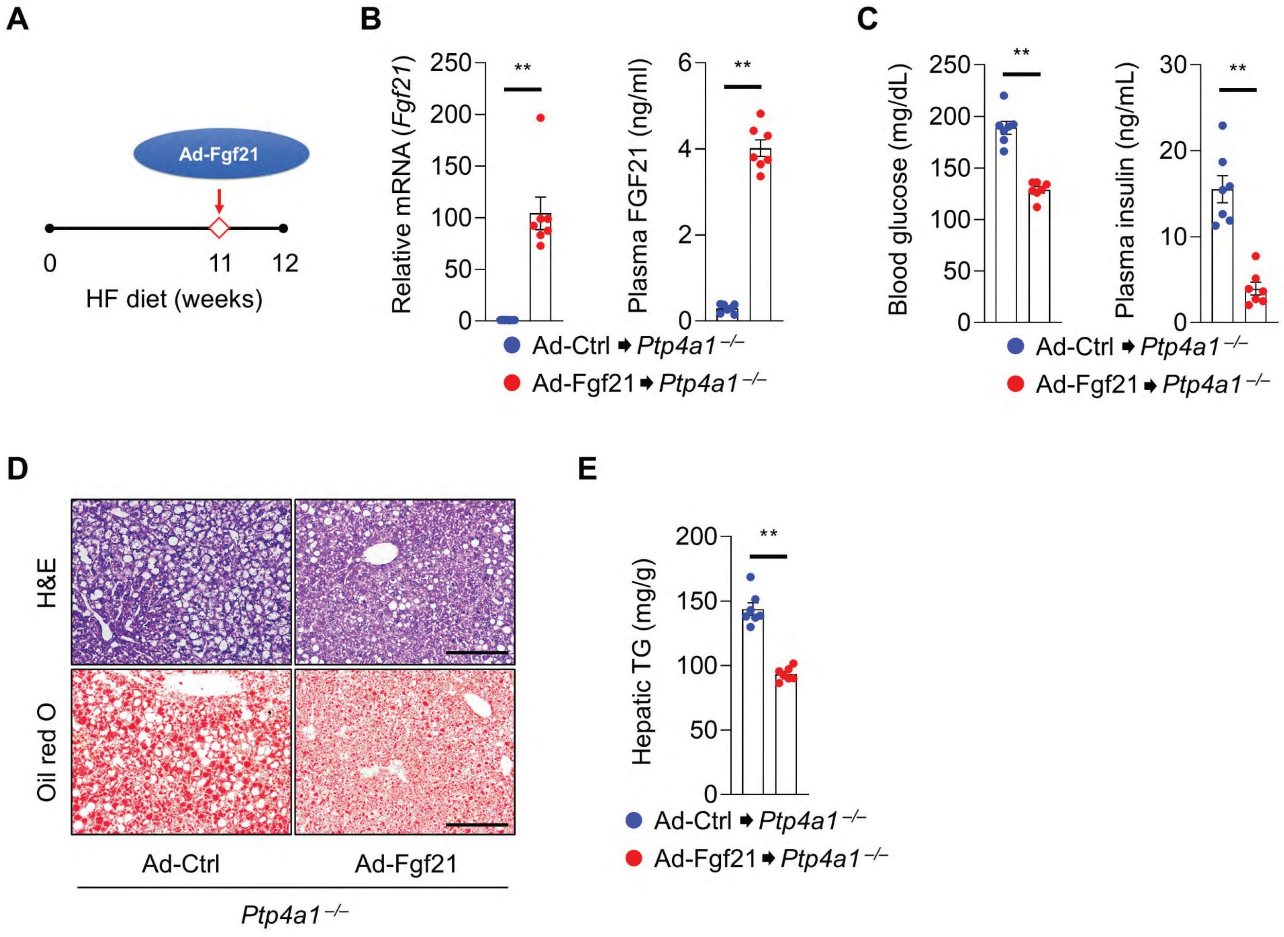
** FGF21 overexpression in *Ptp4a1^-/-^* mice decreases a high-fat (HF) diet-induced hyperglycemia and NAFLD. (A)** The experimental design for administering adenovirus on HF diet-fed *Ptp4a1^-/-^* mice. **(B)** The levels of *Fgf21* mRNA in the liver and FGF21 in plasma of *Ptp4a1^-/-^* mice administrated with Ad-Ctrl or Ad-*Fgf21* after feeding an HF diet (n = 7). **(C)** Blood glucose levels (left) and plasma insulin levels (right) in *Ptp4a1^-/-^* mice administrated with Ad-Ctrl or Ad-*Fgf21* after feeding an HF diet (n = 7). **(D)** The representative images for hematoxylin&eosin (H&E) staining and oil red O staining in the liver sections of *Ptp4a1^-/-^* mice administrated with Ad-Ctrl or Ad-*Fgf21* after feeding an HF diet (n = 7). Scale bar, 200 μm. **(E)** Hepatic triglyceride (TG) levels in *Ptp4a1^-/-^* mice administrated with Ad-Ctrl or Ad-*Fgf21* after feeding an HF diet (n = 7). Data are presented as the mean ± standard error of the mean. **P < 0.01 (two‐tailed Student's t‐test for B (left); Mann-Whitney U test for B (right), C, and E).

Next, we tested whether FGF21 overexpression in *Ptp4a1^-/-^* mice could ameliorate an HF diet-induced hyperglycemia and NAFLD. *Ptp4a1^-/-^* mice fed an HF diet for 11 weeks were administrated with adenovirus (Ad)-Ctrl or Ad-*Fgf21* through the tail vein and kept both groups on an HF diet for an additional one week **(Figure 6A)**. The levels of hepatic *Fgf21* mRNA and plasma FGF21 were markedly increased in the Ad-*Fgf21* group compared to the Ad-Ctrl group **(Figure 6B)**. Overexpression of FGF21 reduced blood glucose levels and plasma insulin levels in *Ptp4a1^-/-^* mice fed an HF diet **(Figure 6C)**. In addition, Ad-*Fgf21* administration decreased the accumulation of lipids in the liver **(Figure 6D-E)**.

### AAV-aat-*Ptp4a1* delivery ameliorates HF diet-induced hyperglycemia and NAFLD in WT mice

**Figure 7:**
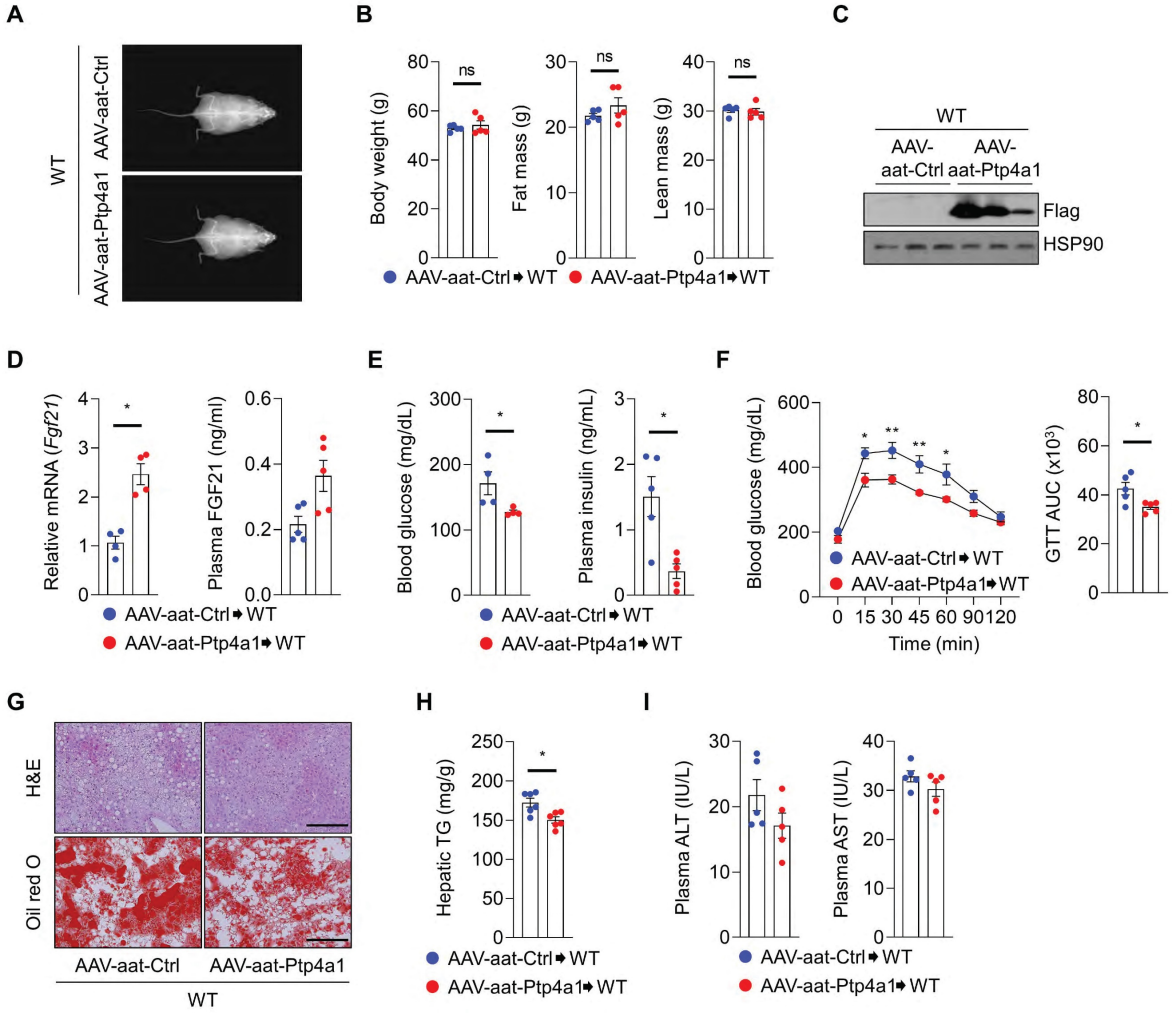
** Liver-specific PTP4A1 overexpression reduces hyperglycemia and NAFLD after feeding a high-fat (HF) diet in wild-type (WT) mice. (A and B)** Representative images (A) and the graphs (B) for body weight, fat, and lean mass of WT mice administrated with adeno-associated virus (AAV)-aat-control (Ctrl) or AAV-aat-*Ptp4a1* after feeding an HF diet for 12 weeks in dual-energy X-ray absorptiometry analysis (n = 5). **(C)** Immunoblot analysis in the liver lysates of WT mice administrated with AAV-aat-Ctrl or AAV-aat-*Ptp4a1*. HSP90 was used as a loading control. **(D)** The levels of *Fgf21* mRNA in the liver (n = 4) and FGF21 in plasma (n = 5) of WT mice administrated with AAV-aat-Ctrl or AAV-aat-*Ptp4a1* after feeding an HF diet.** (E)** Blood glucose levels (left, n = 4) and plasma insulin levels (right, n = 5) in WT mice administrated with AAV-aat-Ctrl or AAV-aat-*Ptp4a1* after feeding an HF diet. **(F)** Glucose tolerance test (GTT) and the area under the curve (AUC) of GTT in WT mice administrated with AAV-aat-Ctrl or AAV-aat-*Ptp4a1* after feeding an HF diet (n = 5). **(G)** The representative images for hematoxylin&eosin (H&E) staining and oil red O staining in the liver sections of WT mice administrated with AAV-aat-Ctrl or AAV-aat-*Ptp4a1* after feeding an HF diet (n = 4). Scale bar, 200 μm. **(H)** Hepatic triglyceride (TG) levels in WT mice administrated with AAV-aat-Ctrl or AAV-aat-*Ptp4a1* after feeding an HF diet (n = 6). **(I)** The levels of alanine aminotransferase (ALT) and aspartate transaminase (AST) in plasma of WT mice administrated with AAV-aat-Ctrl or AAV-aat-*Ptp4a1* after feeding an HF diet (n = 5). Data are presented as the mean ± standard error of the mean. *P < 0.05, n.s., not significant (Mann-Whitney U test for B, D (right), E, F (right), H, and I; two‐tailed Student's t‐test for D (left); two-way ANOVA for F (left)).

Next, we investigated whether hepatic PTP4A1 overexpression by AAV-aat-*Ptp4a1* could reveal metabolic benefits, including improved glucose homeostasis and NAFLD in WT mice fed an HF diet. After an HF diet for 12 weeks, DEXA analysis revealed that WT mice injected with AAV-aat-Ctrl and AAV-aat-*Ptp4a1* showed similar BW, fat mass, and lean mass **(Figure 7A-B)**. AAV-aat-*Ptp4a1* administration highly expressed Flag-PTP4A1 in the liver tissue in WT mice fed an HF diet for 12 weeks **(Figure 7C)**. Hepatic PTP4A1 overexpression increased mRNA levels of hepatic *Fgf21* and plasma FGF21 **(Figure 7D)**. AAV-aat-*Ptp4a1* administration diminished blood glucose and plasma insulin levels compared to AAV-aat-Ctrl in WT mice after feeding an HF diet for 12 weeks **(Figure 7E)**. Improved glucose tolerance was identified in AAV-aat-*Ptp4a1* injected WT mice compared to AAV-aat-Ctrl injected mice; however, the insulin sensitivity was comparable between the two groups **(Figure 7F and S8)**. AAV-aat-*Ptp4a1* significantly reduced lipid accumulation in the liver of WT mice fed an HF diet without the difference in the levels of plasma ALT and AST **(Figure 7G-I)**.

## Discussion

NAFLD is, to date, the most common chronic liver disease leading to a significant health problem worldwide, which affects up to 30% of adults in the general population and 70% of patients with type 2 diabetes 30, 31. Despite understanding the epidemiology and the pathogenic mechanisms in the progress of NAFLD, there are no approved pharmacological therapies for NAFLD 32, providing the need for a novel therapeutic target for the treatment of NAFLD. In the present study, we provided PTP4A1 as a novel regulator in the pathogenesis of NAFLD. PTP4A1 ameliorated hepatosteatosis through the activation of the transcription factor CREBH/FGF21 axis. Liver-specific PTP4A1 expression, followed by augmentation of FGF21 levels, reduced HF diet-induced hepatosteatosis and hyperglycemia in WT mice. Thus, controlling PTP4A1 may be a potential candidate for therapeutic strategy against hepatosteatosis-related diseases.

Protein tyrosine kinases and phosphatases control the phosphorylation of tyrosine residues within proteins that regulate physiological signaling cascades in cells 33. Disruption of the regulation of protein phosphorylation is closely associated with various diseases, including cancers, autoimmune diseases, cardiovascular diseases, and metabolic diseases 34, 35. Recently, protein tyrosine phosphatase 1B (PTP1B), a classical non-transmembrane tyrosine phosphatase, is emerging as a critical regulator in type 2 diabetes, obesity, and liver diseases, suggesting a promising therapeutic target in metabolic disorders 36. PTP1B-null mice resisted Fas-induced extensive hepatocyte apoptosis and promoted hepatocyte proliferation in response to liver damage 36. Thus, PTP1B deficiency in mice revealed a rapid and synchronized compensatory liver regeneration after partial hepatectomy 37. In contrast, PTP4A1 is significantly induced during liver regeneration and is required for proper timing of liver regeneration after partial hepatectomy with modulating expression of cell cycle regulators 10. PTP4A1 constitutes a unique subfamily of protein tyrosine phosphatases and plays a pivotal role during cell development and tissue regeneration 33. Interestingly, both PTP1B and PTP4A1 localized to the endoplasmic reticulum (ER) in non-mitotic cells 38. Based on the opposite phenotype and co-localization in the subcellular compartment between PTP1B and PTP4A1, the beneficial potential of PTP4A1 in metabolic disorders such as NAFLD might be considerable. It may be necessary to test whether PTP4A1 could antagonize PTP1B in various cellular responses.

A previous report verified that PTP4A1 physically interacted with the ATF5/7 containing a basic helix-loop-helix leucine zipper (bZIP) domain 8. In the current study, we proved that PTP4A1 physically interacted with CREBH, a transcription factor containing the bZIP domain, and enhanced the transcriptional activity of CREBH on the *Fgf21* gene. PTP4A1, but not in mutant PTP4A1, synergistically increased CREBH-mediated luciferase activity in an FGF21-luciferase reporter assay. A previous report revealed that proteolytic cleavage of CREBH was modulated by glycogen synthase kinase 3β-mediated phosphorylation 39. As PTP4A1 is localized in ER, plasma membrane, and nucleus, PTP4A1 may enhance both the transcriptional activity of CREBH in the nucleus and proteolytic cleavage of CREBH in ER. Indeed, we verified the synergistic effects in CREBH(N) or CREBH(F) (data not shown) with PTP4A1 in the FGF21 luciferase assay and confirmed interaction in CREBH(N) or CREBH(F) with PTP4A1. Consistent with a previous report 40, a decreased CREBH activity in *Ptp4a1^-/-^* mice may result in the down-regulation of fatty acid oxidation-related gene expressions, including *Pparα* and *Cpt1a*. In a future study, the identification of the PTP4A1-mediated dephosphorylation site of CREBH would be evaluated to understand detailed mechanisms.

FGF21 is an inducible metabolic hormone by fasting or stress, produced mainly in the liver, and regulates glucose and lipid homeostasis via a heterodimeric receptor complex, FGF receptor 1 and β-klotho 41. FGF21 derivatives or FGF21 receptor agonists have been tried as therapeutic agents for various metabolic diseases, including type 2 diabetes, obesity, and NAFLD, for the past decade 21. However, the limitations of FGF21 as a clinically valuable medicine include its poor pharmacokinetics and biophysical properties 41. The current study provided an option to adopt FGF21 therapy in metabolic diseases. Although the oncogenic property of PTP4A1 overexpression and the restricted beneficial effects of PTP4A1-mediated FGF21 remain a hurdle to developing a therapeutic target, PTP4A1-mediated CREBH activation might be considered one of the strategies to elevate FGF21 levels in metabolic disorders, including NAFLD.

In the current study, we identified that *Ptp4a1^-/-^* mice fed an HF diet revealed hyperglycemia and fatty liver compared to controls. In GTT and ITT analysis, *Ptp4a1^-/-^* mice fed an HF diet showed impaired glucose tolerance and insulin sensitivity. However, hepatic glucose output in the clamp study and glucose productions in the pyruvate and glycerol tolerance test were similar between WT and *Ptp4a1^-/-^* mice after feeding an HF diet. The mRNA levels of gluconeogenic genes were not different between the two groups. In addition, glucose uptakes by skeletal muscle and eWAT in the 2-DG uptake assay were similar between the two groups. The levels of phosphorylated Akt in the liver, skeletal muscle, and eWAT were comparable between the two groups (data not shown). Therefore, we could not conclude that PTP4A1 depletion in mice fed an HF diet develops insulin resistance. Instead of insulin resistance, we guessed that PTP4A1 deficiency in mice induced fat accumulation in the liver by feeding an HF diet or fasting, followed by decreased GLUT2 translocation on the hepatocyte surface, leading to hyperglycemia.

Previous reports have shown an HF diet-induced GLUT2 internalization in rat and mouse hepatocytes 16, 42. Also, hepatic GLUT2 location is regulated by feeding states; GLUT2 levels with the insulin receptor in plasma membrane fractions from rat livers are down-regulated after feeding 43. In the current study, we also verified that increased fat accumulation in hepatocytes reduced the levels of GLUT2 on the plasma membrane. Primary hepatocytes from *Ptp4a1^-/-^* mice revealed significant differences in fat accumulation and surface GLUT2 levels compared to WT controls. Although increased fat content is inversely correlated with surface GLUT2 levels in hepatocytes, the detailed mechanism of down-regulating the GLUT2 levels on the plasma membrane of hepatocytes by PTP4A1 deficiency will be clarified in a further study.

In summary, our results establish hepatic PTP4A1-mediated activation of the CREBH/FGF21 axis as a novel therapeutic strategy in NAFLD and hyperglycemia. Lacking PTP4A1 in mice fed an HF diet exhibited hepatosteatosis and hyperglycemia, which were recovered by liver-specific PTP4A1 or systemic FGF21 overexpression. Finally, we proved that liver-specific PTP4A1 expression ameliorated HF diet-induced hepatosteatosis and hyperglycemia in WT mice. Therefore, modulating hepatic PTP4A1 may be a potential therapeutic target against hepatosteatosis-related diseases.

## Materials and Methods

### Animal experiments

This study followed the Guidelines on the Care Use of Laboratory Animals (National Institutes of Health Publication no. 85-23, revised 1996). Animal study protocols were approved by the Institutional Animal Care and Committee of the Korea Research Institute of Bioscience and Biotechnology (KRIBB-AEC-18203). *Ptp4a1^-/-^* mice were generated using the CRISPR/Cas9 system and backcrossed onto a C57BL/6 background. The primers used for *Ptp4a1* genotyping were as follows: forward 5'-TCCGCAGGCTGCCTCCTCTC-3' and reverse 5'-CAGAACAGTAGCAACAAAAT-3'. Mice were kept in a controlled environment with a 12-hour light/dark cycle in a specific pathogen-free facility and fed an NC diet and water for the study. To investigate the effects of PTP4A1 on hepatic steatosis, 8-week-old male mice were given an HF diet (60% calories from fat; D12492, Research DIET) for 12 weeks. Mouse body composition was identified using DEXA (Lunar, GE Lunar Corp.). Body fat and the bone area were demonstrated with a DEXA scan using a dedicated densitometer. After the study, animals were anesthetized using isoflurane inhalation (3%) plus 1 L/min O_2_ and euthanized by exsanguination.

### Measurement of metabolites

Plasma insulin was measured using a mouse insulin enzyme-linked immunosorbent assay (ELISA) kit (Crystal Chem). Plasma AST and ALT were analyzed using a colorimetric assay kit (BioVision). Plasma FGF-21 was measured using a mouse/rat FGF-21 ELISA kit (R&D systems), and hepatic glucose was determined using a glucose assay kit (Sigma). Hepatic TG was measured by Triglyceride Determination Kit (Sigma). 2-DG uptake of mice was analyzed using a 2-DG uptake measurement kit (Cosmo Bio Co.). Blood glucose levels were determined from tail vein blood using an Accu-Check Active blood glucose meter (Roche).

### Tolerance test

Mice were starved for 16 hours and then given an intraperitoneal injection with glucose (1 g/kg BW on HF diet, 2 g/kg BW on NC diet, Sigma), pyruvate (1 g/kg BW on HF diet, 1.5 g/kg BW on NC diet, Sigma), or glycerol (1 g/kg BW on HF diet, 2 g/kg BW on NC diet, Sigma). Mice were starved for six hours and then injected intraperitoneally with mouse insulin (1 unit/kg BW on HF and NC diet, Sigma). Blood glucose levels were measured from tail vein blood collected at the designated times.

### Virus

Liver-specific adeno-associated virus (AAV8.2‐hAAT‐mFlagPTP4A1‐pA) was purchased from Sirion (Martinsried, Germany). For PTP4A1 overexpression in the liver, mice were injected with a concentration of 2.0×10^11^ vg AAV per mouse through the tail vein. PTP4A1 expression levels were determined by immunoblot analysis.

Recombinant adenoviruses expressing FGF21 or PTP4A1 were generated using the AdEasy adenoviral vector system described previously 44. Recombinant adenoviruses were expanded in HEK 293AD cells and purified using an Adeno-X Maxi Purification kit (631533, Clontech). For animal experiments, mice were injected with recombinant adenovirus (1×10^8^ plaque-forming unit per mouse) intravenously through the tail vein. Adenovirus-mediated gene expressions were analyzed by quantitative real time polymerase chain reaction (qRT-PCR) and ELISA.

The lentiviral vector of shPTP4A1 (NM_003463.3-1388s21c1) was purchased from Sigma-Aldrich. Overexpression of PTP4A1 in Hep3B was achieved by lentivirus produced in the pLVX-EF1α-IRES-Puro lentiviral vector (Clontech). Lentivirus-infected cells were selected by puromycin (#P-1033; AG Scientific), and the expression of PTP4A1 was identified by qRT-PCR.

### Primary hepatocytes isolation and cell culture

Primary hepatocytes were prepared from male mice at 8-10 weeks. Livers were perfused with prewarmed liver perfusion medium (17701-038, Life Technologies) followed by liver digest medium (17703-034, Life Technologies). Isolated hepatocytes (5 × 10^5^ cells/well) were placed on Primaria 6-well plates (353846, Corning) and cultured in medium 199 (10-060-CV, Sigma) supplemented with 10% fetal bovine serum (16000-044, Gibco), 20 nM insulin (I6634, Sigma), and 100 nM dexamethasone (D4902, Sigma) under 95% humidified air and 5% CO2. Hep3B and HEK293T cells were grown in DMEM (SH30243.01, HyClone) supplemented with 10% FBS at 37 °C under 95% humidified air and 5% CO2.

### Luciferase reporter gene assay

Hep3B cells were seeded in 48-well plates (4 × 10^4^ cells/well) using DMEM and 10% FBS. Cells were transfected with 50 ng of luciferase reporter, 100 ng of effector, and 5 ng of pRL-TK (*Renilla* TK) plasmids using 0.5 μl of the transfection reagent Lipofectamine 2000 (Invitrogen). Cells were lysed and moved into 96-well plates 48 hours later for luciferase assays using the Dual-Luciferase Reporter Assay kit (E1960, Promega). Luciferase activity was measured on a fluorescence spectrophotometer (GloMax 96, Promega), and relative transcriptional activity was normalized by the Renilla activity.

### Hyperinsulinemic-euglycemic clamping

Hyperinsulinemic-euglycemic clamping was executed as previously described 45. Briefly, *Ptp4a1^-/-^* mice and WT were fed an HF diet, and then a hyperinsulinemic-euglycemic clamp was subjected. After an overnight fast, basal glucose levels were preserved by 20% glucose infused at different rates. Plasma glucose levels were consistently measured every 20 minutes. The insulin clamp was initiated with a primed-continuous infusion of 0.5 U/ml insulin. Basal and insulin-stimulated whole-body glucose uptake was measured through a consecutive input of ^3^H-glucose (NET-331C-2, PerkinElmer) for two hours before clamping (0.05 μCi/min) and during clamping (0.1 μCi/min), respectively. 2-deoxy-_D_-1-^14^C glucose (NEC-495-1, PerkinElmer) was infused to measure insulin-stimulated glucose uptake 75 minutes later. Plasma samples were dissolved in ZnSO_4_ (83265, Sigma) and Ba(OH)_2_ (B4059, Sigma), dried, resuspended in water, and detected with ^3^H-glucose and 2-deoxy-_D_-1-^14^C concentrations in scintillation fluid.

### Histology and Immunohistochemistry

Liver pieces were fixed in 10% (v/v) phosphate-buffered formalin solution overnight at room temperature and then placed in 30% sucrose overnight at 4 °C. The fixed tissues were embedded in paraffin or OCT (3801480, Leica) and sectioned. H&E staining was applied to frozen and paraffin-embedded tissue sections. Oil-red O staining was used on frozen tissue sections. After staining, images were captured under a light microscope (BX53F2, Olympus Corp).

For immunostaining, liver sections were permeabilized with 0.05% (v/v) Triton X-100, blocked with 10% (v/v) chicken serum and 1% (w/v) BSA, and incubated with anti-CD45 (ab10558, Abcam) or anti-GLUT2 (ab54460, Abcam) antibody with 1% (w/v) BSA in TBS for overnight at 4 °C. Slides were visualized using biotinylated secondary antibodies with a 3,3′-diaminobenzidine substrate (PK-6100, Vector Laboratories). Nuclei were stained with hematoxylin. Immuno-staining images were captured using fluorescent and light microscopes (BX53F2, Olympus Corp).

### Immunoprecipitation and Western blotting

Mouse tissues and cells were harvested and dissolved in RIPA buffer (50 mM Tris-HCl, 150 mM NaCl, 1 mM EDTA, 0.5% sodium deoxycholate, 1% Triton-X-100, 0.5% NP-40, pH 7.6) or NP-40 buffer (50 mM Tris-HCl, 150 mM NaCl, 1% NP-40, pH 7.4) containing protease and phosphatase inhibitor cocktail (GenDEPOT, Huston). Immunoprecipitation was performed with anti-Flag (F1804, Sigma) antibody overnight at 4 °C, followed by addition to protein G-Sepharose beads (Upstate Biotechnology) for two hours at 4 °C. Liver nuclear extracts were prepared as described previously 27. Protein lysates were performed to Western blotting with the following primary antibodies [rat anti-HA (3F10, Roche), rabbit anti-GLUT2 (ab54460, Abcam), rabbit anti-β-actin (AbC-2004, Abclon), mouse anti-FLAG (F1804, Sigma), mouse anti-HSP90α/β (sc-13119, Santacruz), rabbit anti-CREBH (EWS101, Kerafast), mouse anti-Lamin B1 (sc-377001, Santacruz), goat anti-PTP4A1(EB06456, Everest), and rabbit anti-GSK3 β (#12456, Cell Signaling Technology)]. The membranes were incubated with primary antibodies followed by the horseradish peroxidase-conjugated secondary antibodies (rat: 31470, rabbit: 31464, Thermo Fischer Scientific; mouse: AbC-5001, AbClon) for one hour at room temperature. Immuno-reactive bands were visualized using a chemiluminescent substrate (RPN2106, GE).

### RNA isolation and qRT-PCR

Total RNA was isolated using TRIZOL Reagents (15596026, Life Technologies) according to the manufacturer's recommendation. Complementary DNA was synthesized from target RNA using the M-MLV Reverse Transcriptase kit (N1705, C1101, N2515, U1518; Promega). The qRT-PCR was performed using the quantiMix SYBR kit (QS105; PKT Co.). The mRNA expression levels were normalized using human GAPDH or mouse 18s expression. The qRT-PCR primers are listed in Supplementary Table 1.

### Flow cytometry

Isolated primary hepatocytes were blocked with rat IgG antibody (1 μg/ml, MAB005, R&D systems) in phosphate buffered saline (PBS) containing 1% (w/v) BSA for 15 minutes at room temperature. Next, cells were washed three times and incubated with the rat anti-GLUT2-allophycocyanin (APC) antibody (1 μg/ml, FAB1440A, R&D systems), mouse anti-asialoglycoprotein receptor 1 (ASGR1) (1 μg/ml, AF2755, R&D systems) with Alexa Flour 488 conjugated anti-mouse IgG antibody in PBS containing 1% (w/v) BSA. For lipids staining or glucose uptake assay, primary hepatocytes and Hep3B were incubated with BODIPY (20 μM, D-3922, Invitrogen) or 2-NBDG (500 μM, N13195, Invitrogen), respectively. After incubation, cells were washed three times and promptly analyzed on a FACSCalibur (BD Immunocytometry System).

### Chromatin immunoprecipitation (ChIP) assay

Hep3B cells (1 × 10^7^) were processed using the EZ-Chip^TM^ Kit (17-371; Millipore, Darmstadt, Germany) according to the manufacturer's instructions. Briefly, genomic DNA was crosslinked with 1% formaldehyde and fragmented into 500 ± 100 bp fragments by sonicating for 10 seconds with 5 μm/wave ten times. Soluble chromatin was incubated overnight with each antibody (HA antibody and human RNA polymerase II antibody). Immunoprecipitated DNA fragments were amplified and quantified by qRT-PCR using specific primers to the *FGF21* gene promoter (CREBH binding region) and *GAPDH* gene promoter. The primers used for the *FGF21* gene were forward 5'-CAGGCTGCCCTTGCCACGATG-3' and reverse 5'-ATACCCAGACAGGCCCGCCCA-3'. The primers used for the *GAPDH* gene were forward 5'-TACTAGCGGTTTTACGGGCG -3' and reverse 5'-TCGAACAGGAGGAGCAGAGAGCGA-3'.

### Statistics

Data are expressed as the mean ± standard error of the mean. We subjected two‐tailed Student's *t*-tests, one-way ANOVA, two-way ANOVA, the Mann-Whitney U test, and linear regression analysis to test statistical significance where appropriate. Linear correction index *R* square and *P* value were calculated. Statistical tests are described in Figure Legends for each experiment. *P* values less than 0.05 were considered significant.

## Supplementary Material

Supplementary figures and table.

## Abbreviations

AAT: alpha-1-antitrypsin
AAV: adeno-associated virus
Ad: adenovirus
ALT: alanine aminotransferase
ASGR1: asialoglycoprotein receptor 1
AST: aspartate transaminase
ATF5/7: activating transcription factor 5/7
BSA-OA: bovine serum albumin-oleic acid
BW: body weight
bZIP: basic helix-loop-helix leucine zipper
CD45): cluster of differentiation 45
CREBH: cyclic adenosine monophosphate-responsive element-binding protein H
Ctrl: control
DEXA: dual-energy X-ray absorptiometry
2-DG: 2-deoxyglucose
ELISA: enzyme-linked immunosorbent assay
ER: endoplasmic reticulum
eWAT: epididymal white adipose tissue
FGF21: fibroblast growth factor 21
GLUT2: glucose transporter 2
GlyTT: glycerol tolerance test
GTT: glucose tolerance test
H&E: hematoxylin and eosin
HEK293T: human embryonic kidney 293T
HF: high-fat
HGP: hepatic glucose production
HOMA-IR: homeostatic model assessment-insulin resistance
ITT: insulin tolerance test
NAFLD: non-alcoholic fatty liver disease
2-NBDG: 2-(N-(7-Nitrobenz-2-oxa-1,3-diazol-4-yl)Amino)-2-Deoxyglucose
NC: normal chow
PBS: phosphate buffered saline
PKB: protein kinase B
PPARα: peroxisome proliferator-activated receptor α
PTP1B: protein tyrosine phosphatase 1B
PTP4A1: protein tyrosine phosphatase type IVA 1
PRL-1: phosphatase of regenerating liver-1
PTT: pyruvate tolerance test
qRT-PCR: quantitative real time polymerase chain reaction
shRNA: short hairpin RNA
TG: triglyceride
USF1: upstream stimulatory factor 1
WT: wild-type
