# Supplementary material for: Hepatic PTP4A1 ameliorates high-fat diet-induced hepatosteatosis and hyperglycemia by the activation of the CREBH/FGF21 axis
Source: Theranostics. 2023 Jan 22;13(3):1076–90. doi: 10.7150/thno.79434 (PMC9925322; doi:10.7150/thno.79434)
Supplement: Supplementary file 1 — Supplementary figures and table. [file thnov13p1076s1.pdf]

Hepatic PTP4A1 ameliorates high-fat diet-induced hepatosteatosis and hyperglycemia by the activation of the CREBH/FGF21 axis

Byungtae Hwang[1,#], Min-Gi Kwon[1,4,#], Min Ji Cho[1], Nam-Kyung Lee[1], Jangwook Lee[1], Jeong Woong Lee[1], Kyoung-Jin Oh[2], Kwang-Hee Bae[2], Jung Hwan Hwang[3], Jeong-Ki Min[1,4,*], Jong-Gil Park[1,4,*]

[1]Biotherapeutics Translational Research Center, Korea Research Institute of Bioscience & Biotechnology (KRIBB), 125 Gwahak-ro, Yuseong-gu, Daejeon 34141, Republic of Korea.

[2]Metabolic Regulation Research Center, Korea Research Institute of Bioscience & Biotechnology (KRIBB), 125 Gwahak-ro, Yuseong-gu, Daejeon 34141, Republic of Korea.

[3]Laboratory Animal Resource Center, Korea Research Institute of Bioscience & Biotechnology (KRIBB), 125 Gwahak-ro, Yuseong-gu, Daejeon 34141, Republic of Korea.

[4]Department of Bioscience, KRIBB School of Bioscience, Korea University of Science and Technology (UST), 125 Gwahak-ro, Yuseong-gu, Daejeon 34141, Republic of Korea.

[#] These authors contributed equally to this work

[*]Correspondence to Jeong-Ki Min, PhD., Biotherapeutics Translational Research Center, Korea Research Institute of Bioscience & Biotechnology, 125 Gwahak-ro, Yuseong-gu, Daejeon 34141, Republic of Korea; Tel: +82-42-860-4137; Fax: +82-42-860-4149; E-mail: jekmin74@gmail.com

[*]Correspondence to Jong-Gil Park, PhD., Biotherapeutics Translational Research Center, Korea Research Institute of Bioscience & Biotechnology, 125 Gwahak-ro, Yuseong-gu, Daejeon 34141, Republic of Korea; Tel: +82-42-860-4122; Fax: +82-42-860-4149; E-mail: jonggilpark@kribb.re.kr

Supplementary Table 1. qRT-PCR primer sequences

Primer name (mouse)	Primer sequence	
Ptp4a1	Forward	CAACTTACGACACTACTCTT
	Reverse	CAGCAACCAGGTTCTTCACG
Ptp4a2	Forward	TGCTACATATGATAAAGCTCC
	Reverse	CTACTGAACACAGCAGTGCC
Ptp4a3	Forward	CCTATGACAAGACCCCCCTG
	Reverse	CTACATGACGCAGCATCTGG
F4/80	Forward	CTTTGGCTATGGGCTTCCAGTC
	Reverse	GCAAGGAGGACAGAGTTTATCGTG
Mcp1	Forward	AGGTCCCTGTCATGCTTCTG
	Reverse	GCTGCTGGTGATCCTCTTGT
Mip1a	Forward	TTCTCTGTACCATGACACTCTGC
	Reverse	CGTGGAATCTTCCGGCTGTAG
CD11c	Forward	ACACAGTGTGCTCCAGTATGA
	Reverse	GCCCAGGGATATGTTCACAGC
KC	Forward	ACTGCACCCAAACCGAAGTC
	Reverse	TGGGGACACCTTTTAGCATCTT
Foxo1	Forward	TGGTGAACACCATGCCTCAC
	Reverse	CTTCTCCTGGTGGAGGACAC
Pck1	Forward	CTGCATAACGGTCTGGACTTC
	Reverse	CAGCAACTGCCCGTACTCC
G6pc	Forward	CGACTCGCTATCTCCAAGTGA
	Reverse	GTTGAACCAGTCTCCGACCA
Fgf21	Forward	GGAGCTCTCTATGGATCGCCT
	Reverse	TGTAACCGTCCTCCAGCAGC
Srebf1	Forward	CAGCTCAGAGCCGTGGTGA
	Reverse	TTGATAGAAGACCGGTAGCGC
Srebf2	Forward	GTGGAGCAGTCTCAACGTCA
	Reverse	CCGGATGCATGGTAGGTCTC
ChREBP	Forward	CCAGCCTCAAGGTGAGCAAA
	Reverse	CATGTCCCGCATCTGGTCA
Fasn	Forward	GGAGGTGGTGATAGCCGGTAT
	Reverse	TGGGTAATCCATAGAGCCCAG
Scd1	Forward	TTCTTGCGATACACTCTGGTGC
	Reverse	CGGGATTGAATGTTCTTGTCGT
Elov5	Forward	ATGGAACATTTCGATGCGTCA
	Reverse	GTCCCAGCCATACAATGAGTAAG
Dgat2	Forward	GAAGCTGCCCGCAGCGAAAA
	Reverse	TCTTGGGCGTGTTCCAGTCAA

Ppara	Forward	CTGCAGAGCAACCATCCAGA
	Reverse	TGATGACCTGTACGAGCTGC
Acox1	Forward	TCAACAGCCCAACTGTGACT
	Reverse	GGCCGATATCCCCAACAGTG
Cpt1a	Forward	CTCCGCCTGAGCCATGAAG
	Reverse	CACCAGTGATGATGCCATTCT
Cpt2	Forward	CAGCACAGCATCGTACCCA
	Reverse	TCCCAATGCCGTTCTCAAAAT
Hadh	Forward	TCAAGCATGTGACCGTCATCG
	Reverse	TGGATTTTGCCAGGATGTCTTC
Ehhadh	Forward	ATGGCTGAGTATCTGAGGCTG
	Reverse	GGTCCAAACTAGCTTTCTGGAG
Acadvl	Forward	CTACTGTGCTTCAGGGACAAC
	Reverse	CAAAGGACTTCGATTCTGCCC
Acadl	Forward	TCTTTTCCTCGGAGCATGACA
	Reverse	GACCTCTCTACTCACTTCTCCAG
Acadm	Forward	AGGGTTTAGTTTTGAGTTGACGG
	Reverse	CCCCGCTTTTGTCATATTCCG
18s	Forward	CGCCGCTAGAGGTGAAATTC
	Reverse	TTGGCAAATGCTTTCGCT
Primer name (human)	Primer sequence	
PTP4A1	Forward	GTGAAGCAACTTATGACACT
	Reverse	CAGAAGTTGCTTGCTGTTAA
PTP4A2	Forward	AGCTCCACCCCCTAATCAGA
	Reverse	TTGAACGCTCCCCTTCTTTT
PTP4A3	Forward	CGGCAAGGTAGTGGAAGAC
	Reverse	GGCGGATGAACTGGATGG
FGF21	Forward	ATGGATCGCTCCACTTTGACC
	Reverse	GGGCTTCGGACTGGTAAACAT
GAPDH	Forward	CGCCACAGTTTCCCGGAGGG
	Reverse	CCCTCCAAAATCAAGTGGGG

Supplementary Figures and Legends

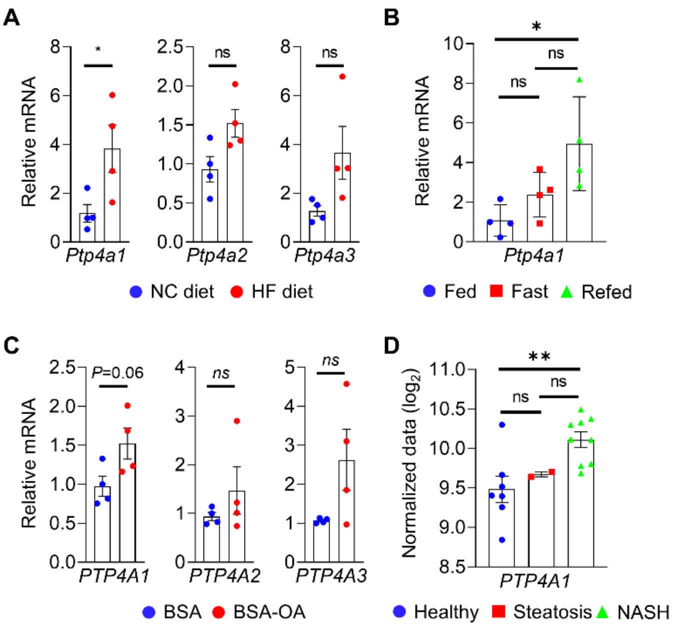

Figure S1. Lacking PTP4A1 in mice exacerbates a high-fat (HF) diet-induced hyperglycemia and NAFLD, related to Figure 1.

(A) The mRNA levels of the *Ptp4a1*, *Ptp4a2*, and *Ptp4a3* in the livers of WT mice fed an HF diet or a normal chow (NC) diet (n = 4). **(B)** The mRNA levels of the *Ptp4a1* in the livers of WT mice on an NC diet in feeding and fasting conditions (n = 4). **(C)** The mRNA levels of the *PTP4A1*, *PTP4A2*, and *PTP4A3* in Hep3B treated with bovine serum albumin (BSA) or BSA-oleic acid (BSA-OA) (n = 4). **(D)** The levels of PTP4A1 gene expression in human liver samples of healthy control, steatosis, and non-alcoholic steatohepatitis (NASH) from the dataset GSE63067. Data are presented as the mean ± standard error of the mean. *$P < 0.05$, **$P < 0.01$, n.s., not significant (two-tailed Student's t-test for A and C; one-way ANOVA for B and D).

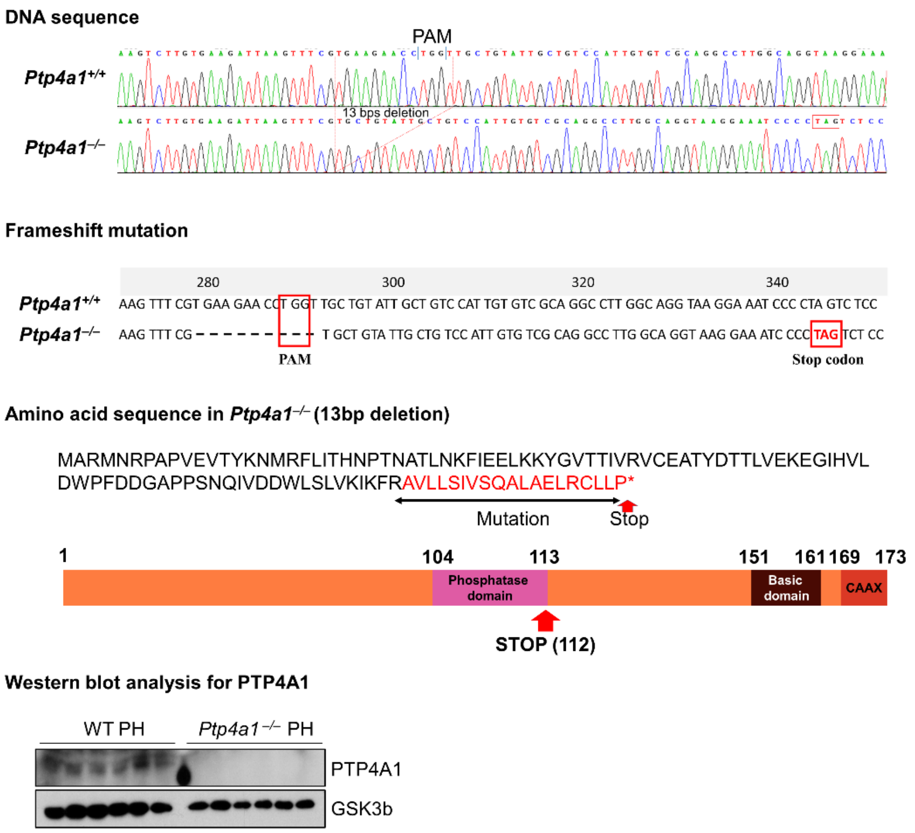

Figure S2. Lacking PTP4A1 in mice exacerbates a high-fat (HF) diet-induced hyperglycemia and NAFLD, related to Figure 1.

Generation of *Ptp4a1*$^{-/-}$ mice using the CRISPR/Cas9 system. Genomic DNA sequences, frameshift mutation-induced first stop codon, predicted premature amino acid sequences, and western blot analysis for PTP4A1 in the lysates of primary hepatocytes from *Ptp4a1*$^{+/+}$ and *Ptp4a1*$^{-/-}$mice. GSK3β was used as a loading control. The protospacer adjacent motif (PAM) sequence (TGG) is indicated in the red box on the genomic DNA sequence.

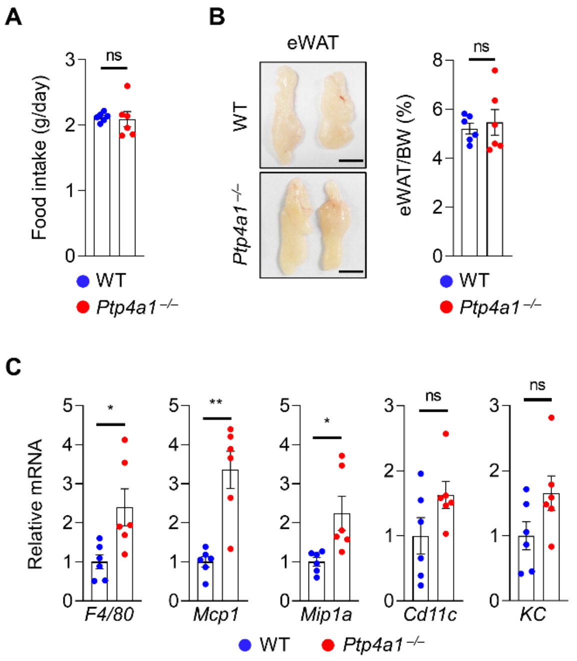

Figure S3. Lacking PTP4A1 in mice exacerbates a high-fat (HF) diet-induced hyperglycemia and NAFLD, related to Figure 1.

(A) Food intake of *Ptp4a1*$^{-/-}$ mice and wild-type (WT) littermates on an HF diet (n = 6). **(B)** The representative images of epididymal white adipose tissue (eWAT) and the ratio of eWAT mass to body weight (BW) in WT and *Ptp4a1*$^{-/-}$ mice after feeding an HF diet for 12 weeks (n = 6). Scale bar, 1 cm. **(C)** The mRNA levels of the *F4/80*, *Mcp1*, *Mip1a*, *Cd11c*, and *KC* in the livers of WT and *Ptp4a1*$^{-/-}$ mice fed an HF diet for 12 weeks (n = 6). Data are presented as the mean ± standard error of the mean. **P* < 0.05, ***P* < 0.01, n.s., not significant (Mann–Whitney U test for A and B; two-tailed Student's t-test for C).

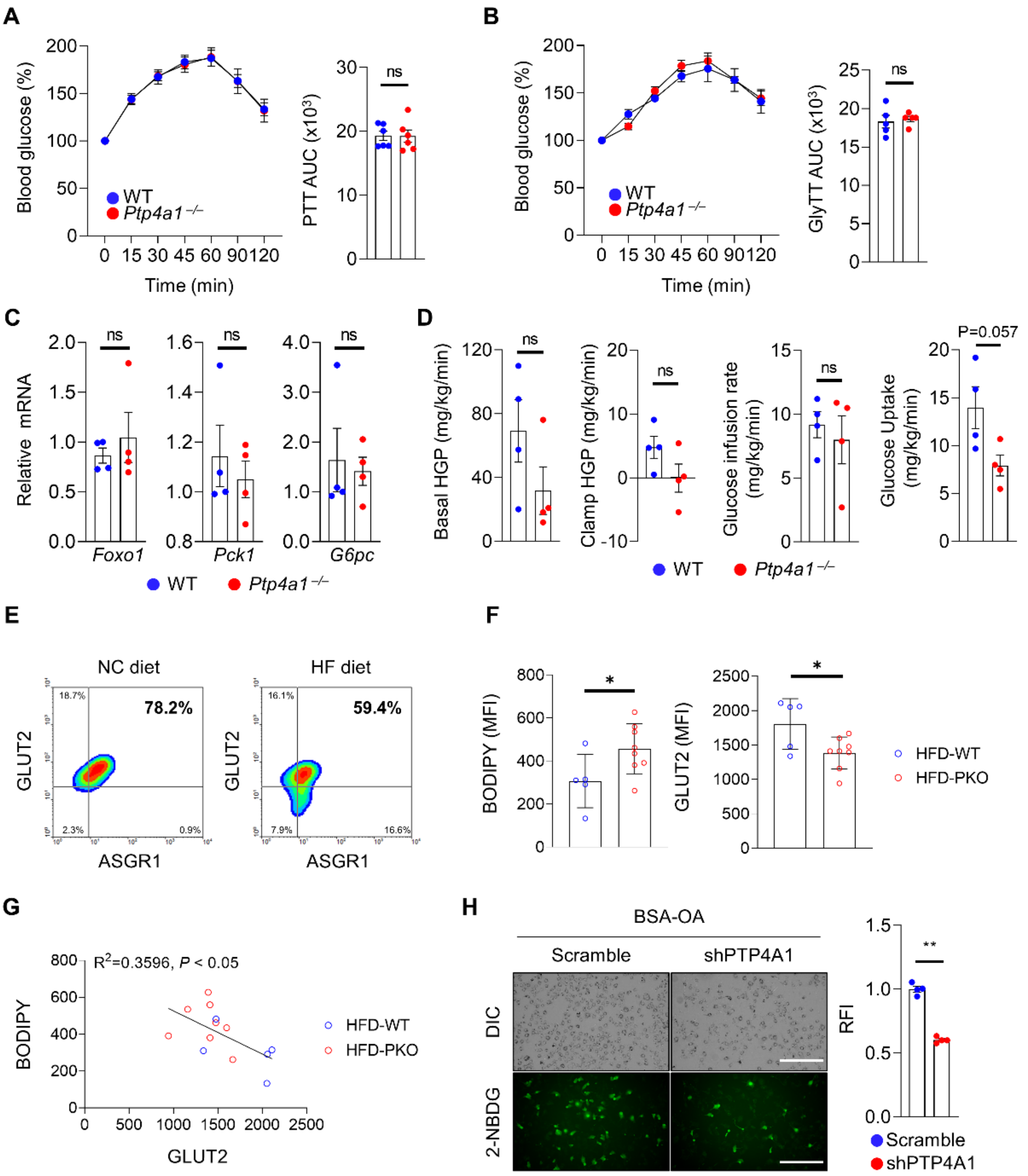

Figure S4. The deficiency of PTP4A1 in mice fed a high-fat (HF) diet reduces glucose uptake by a decrease in GLUT2 on the plasma membrane in hepatocytes, related to Figure 2.

(A) The pyruvate tolerance test (PTT, n = 6) and **(B)** glycerol tolerance test (GlyTT, n = 5) with the area under the curve (AUC) in wild-type (WT) and *Ptp4a1*$^{-/-}$ mice after feeding an HF diet. **(C)** The mRNA levels of the *Foxo1*, *Pck1*, and *G6pc* genes in the livers of WT and *Ptp4a1*$^{-/-}$ mice fed an HF diet for 12 weeks (n = 4). **(D)** The hyperinsulinemic-euglycemic clamp study for basal hepatic glucose production (HGP), clamp HGP, glucose infusion rate, and glucose uptake in WT and *Ptp4a1*$^{-/-}$ mice

fed an HF diet (n = 54). **(E)** FACS analysis after staining glucose transporter 2 (GLUT2)-APC and asialoglycoprotein receptor 1 (ASGR1)-Alexa 488 on primary hepatocytes of WT mice fed a normal chow (NC) or an HF diet. ASGR1 was used as a marker for hepatocytes. Data represent three independent experiments. **(F)** Mean fluorescence intensity (MFI) for the BODIPY and surface GLUT2-APC in primary hepatocytes of WT and *Ptp4a1*$^{-/-}$ mice fed an HF diet by FACS analysis (n = 5–8). **(G)** Linear regression of BODIPY and GLUT2-APC levels in primary hepatocytes (n = 13). **(H)** The 2-NBDG uptake assay after incubation of bovine serum albumin-oleic acid (BSA-OA) on Hep3B treated by PTP4A1-specific shRNA-expressing lentivirus or control lentivirus. Representative images (left) and quantification for relative fluorescence intensity (RFI, right) (n = 4). Scale bar, 500 μm. Data are presented as the mean ± standard error of the mean. $^{**}P < 0.01$, n.s., not significant (Mann–Whitney U test for A, B, and D; two-tailed Student's t-test for C, F, and H; Linear regression for G).

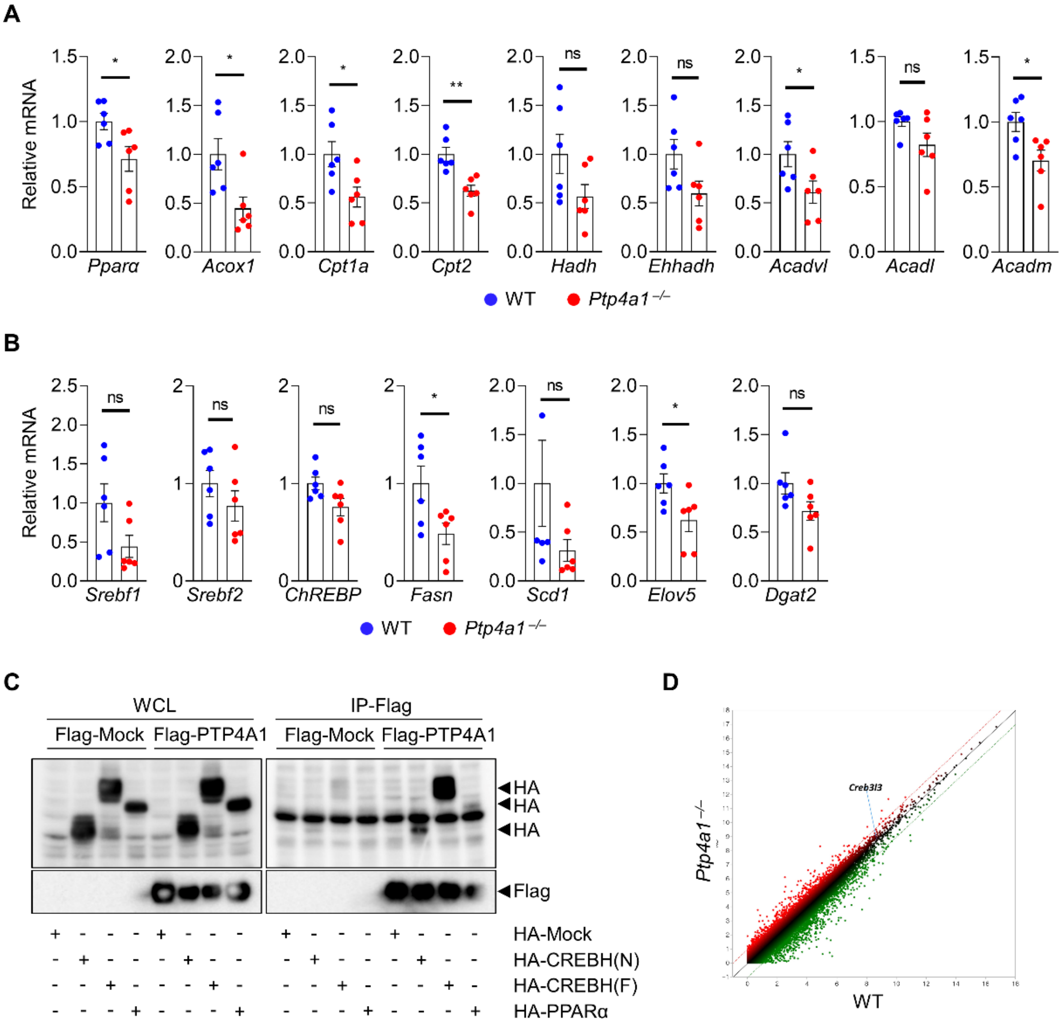

Figure S5. PTP4A1 regulates the expression of FGF21 via the up-regulation of CREBH activity, related to Figure 3.

(A) The mRNA levels of *Pparα*, *Acox1*, *Cpt1a*, *Cpt2*, *Hadh*, *Ehhadh*, *Acadvl*, *Acadl*, and *Acadm* genes in the livers of wild-type (WT) and *Ptp4a1*$^{-/-}$ mice fed an HF diet for 12 weeks (n = 6). **(B)** The mRNA levels of *Srebf1*, *Srebf2*, *ChREBP*, *Fasn*, *Scd1*, *Elov5*, and *Dgat2* genes in the livers of WT and *Ptp4a1*$^{-/-}$ mice fed an HF diet for 12 weeks (n = 6). **(C)** Co-immunoprecipitation assay in HEK 293T transfected by HA-Mock, HA-CREBH(N), HA-CREBH(F), or HA-PPARα with Flag-Mock or Flag-PTP4A1. Flag antibody was used for immunoprecipitation. **(D)** The scatter plot indicates the expression of the *Creb3l3* gene (encoding CREBH) in the liver of WT and *Ptp4a1*$^{-/-}$ mice fed an HF diet for 12 weeks. Data represent three independent experiments. Data are presented as the mean ±

standard error of the mean. *$P < 0.05$, **$P < 0.01$, n.s., not significant (two-tailed Student's t-test for A and B)

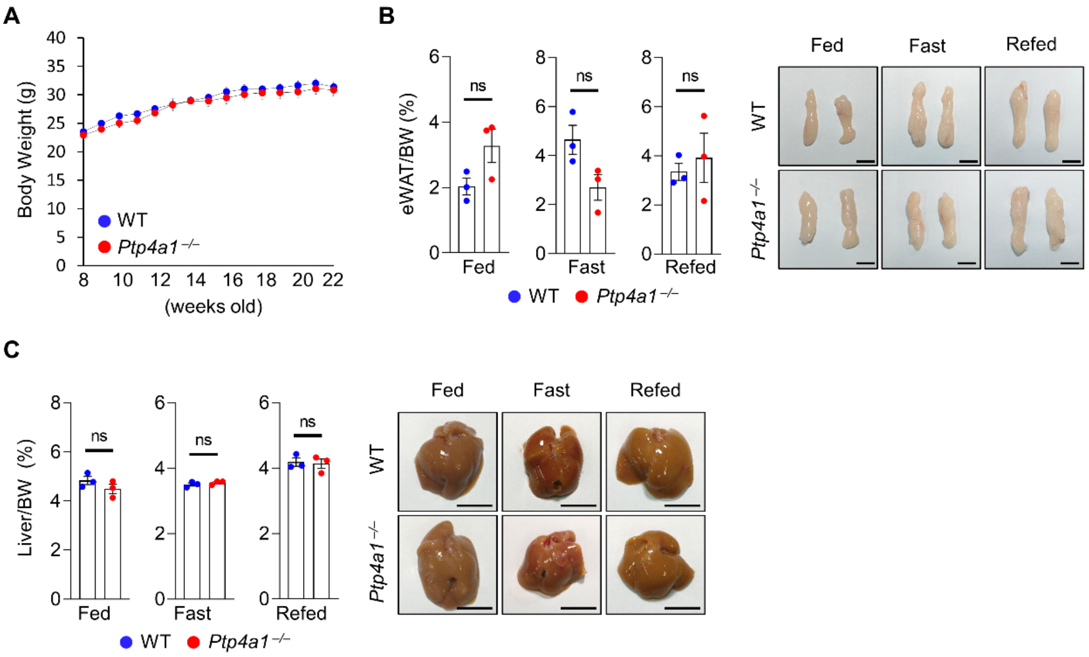

Figure S6. Lacking PTP4A1 in mice increases blood glucose and NAFLD by the down-regulation of FGF21 expression in fasting conditions, related to Figure 4.

(A) Body weight (BW) gain of *Ptp4a1*$^{-/-}$ mice and wild-type (WT) littermates on a normal chow (NC) diet from 8-week-old to 22-week-old (n = 9). **(B)** The ratio of epididymal white adipose tissue (eWAT) mass to BW and the representative images of eWAT in WT and *Ptp4a1*$^{-/-}$ mice after feeding an NC diet at 22-week-old (n = 3). Scale bar, 1 cm. **(C)** The ratio of liver mass to BW and the representative images of the liver in WT and *Ptp4a1*$^{-/-}$ mice after feeding an NC diet at 22-week-old (n = 3). Scale bar, 1 cm. Data are presented as the mean ± standard error of the mean. n.s., not significant (Mann–Whitney U test for B and C).

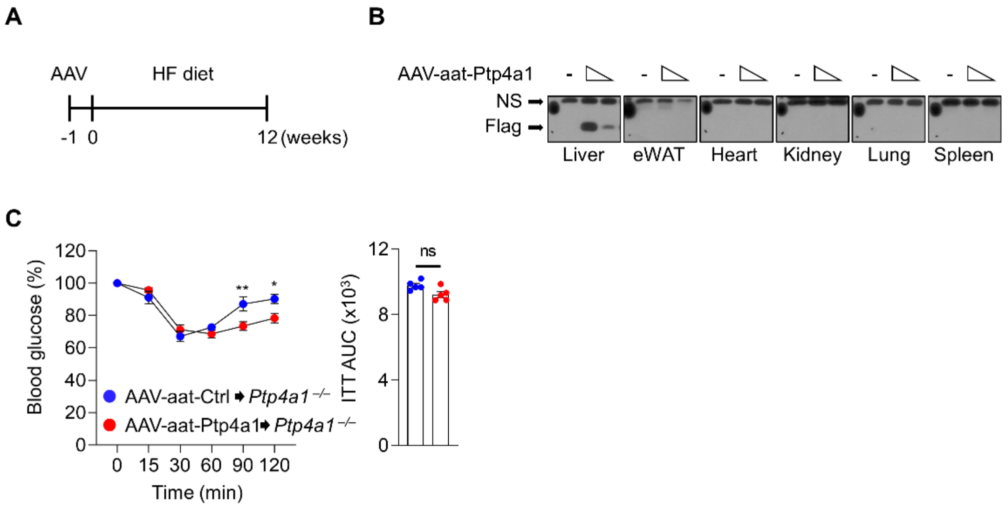

Figure S7. Liver-specific PTP4A1 overexpression ameliorates hyperglycemia and NAFLD in *Ptp4a1*$^{-/-}$ mice fed a high-fat (HF) diet, related to Figure 5.

(A) The experimental design for administering adeno-associated virus (AAV) and HF diet feeding of *Ptp4a1*$^{-/-}$ mice. **(B)** Immunoblot analysis of the lysates from the liver, epididymal white adipose tissue (eWAT), heart, kidney, lung, and spleen in *Ptp4a1*$^{-/-}$ mice injected by AAV-aat-control (Ctrl) or AAV-aat-*Ptp4a1*. NS: the non-specific band. **(C)** Insulin tolerance test (ITT) and the area under the curve (AUC) of ITT in *Ptp4a1*$^{-/-}$ mice administrated with AAV-aat-Ctrl or AAV-aat-*Ptp4a1* after feeding an HF diet (n = 5). Data are presented as the mean ± standard error of the mean. *$P < 0.05$, **$P < 0.01$, n.s., not significant (two-way ANOVA for C (left); Mann–Whitney U test for C (right)).

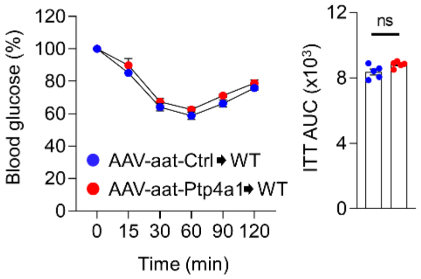

Figure S8. Liver-specific PTP4A1 overexpression reduces hyperglycemia and NAFLD after feeding a high-fat (HF) diet in wild-type (WT) mice, related to Figure 7.

Insulin tolerance test (ITT) and the area under the curve (AUC) of ITT in WT mice administrated with AAV-aat-Ctrl or AAV-aat-*Ptp4a1* after feeding an HF diet (n = 5). Data are presented as the mean ± standard error of the mean. n.s., not significant (Mann–Whitney U test for the AUC graph).
